# Long-Term Simulation of Microgravity Induces Changes in Gene Expression in Breast Cancer Cells

**DOI:** 10.3390/ijms24021181

**Published:** 2023-01-07

**Authors:** Jayashree Sahana, José Luis Cortés-Sánchez, Viviann Sandt, Daniela Melnik, Thomas J. Corydon, Herbert Schulz, Zexi Cai, Katja Evert, Daniela Grimm, Markus Wehland

**Affiliations:** 1 Department of Biomedicine, Aarhus University, 8000 Aarhus, Denmark; 2 Department of Microgravity and Translational Regenerative Medicine, Otto von Guericke University, 39106 Magdeburg, Germany; 3 Research Group “Magdeburger Arbeitsgemeinschaft für Forschung Unter Raumfahrt- Und Schwerelosigkeitsbedingungen” (MARS), Otto Von Guericke University, 39106 Magdeburg, Germany; 4 Department of Ophthalmology, Aarhus University Hospital, 8200 Aarhus, Denmark; 5 Center for Quantitative Genetics and Genomics, Aarhus University, 8000 Aarhus, Denmark; 6 Institute of Pathology, University of Regensburg, 93953 Regensburg, Germany

**Keywords:** breast cancer, spheroids, cytoskeleton, extracellular matrix, focal adhesion, microgravity, PAM signaling

## Abstract

Microgravity changes the gene expression pattern in various cell types. This study focuses on the breast cancer cell lines MCF-7 (less invasive) and MDA-MB-231 (triple-negative, highly invasive). The cells were cultured for 14 days under simulated microgravity (s-µ*g*) conditions using a random positioning machine (RPM). We investigated cytoskeletal and extracellular matrix (ECM) factors as well as focal adhesion (FA) and the transmembrane proteins involved in different cellular signaling pathways (MAPK, PAM and VEGF). The mRNA expressions of 24 genes of interest (*TUBB*, *ACTB*, *COL1A1*, *COL4A5*, *LAMA3*, *ITGB1*, *CD44*, *VEGF*, *FLK1*, *EGFR*, *SRC*, *FAK1*, *RAF1*, *AKT1*, *ERK1*, *MAPK14*, *MAP2K1*, *MTOR*, *RICTOR*, *VCL*, *PXN*, *CDKN1*, *CTNNA1* and *CTNNB1*) were determined by quantitative real-time PCR (qPCR) and studied using STRING interaction analysis. Histochemical staining was carried out to investigate the morphology of the adherent cells (ADs) and the multicellular spheroids (MCSs) after RPM exposure. To better understand this experimental model in the context of breast cancer patients, a weighted gene co-expression network analysis (WGCNA) was conducted to obtain the expression profiles of 35 breast cell lines from the HMS LINCS Database. The qPCR-verified genes were searched in the mammalian phenotype database and the human genome-wide association studies (GWAS) Catalog. The results demonstrated the positive association between the real metastatic microtumor environment and MCSs with respect to the extracellular matrix, cytoskeleton, morphology, different cellular signaling pathway key proteins and several other components. In summary, the microgravity-engineered three-dimensional MCS model can be utilized to study breast cancer cell behavior and to assess the therapeutic efficacies of drugs against breast cancer in the future.

## 1. Introduction

The 2020 GLOBOCAN (Global Cancer Observatory) survey estimated a total of 2.3 million new cases of and 684,996 deaths caused by female breast cancer (BC) worldwide [1]. BC is heterogeneous and is broadly classified into three types: luminal (luminal A, the most common type, and luminal B), HER2+ (the rarest) and triple-negative BC (TNBC). These BC types differ in prognosis, progression, metastasis and therapeutic response. Local options for treatment are surgery and radiation. Systemic treatments comprise chemotherapy, hormone therapy, immunotherapy and drug-targeted treatment strategies. Various drugs are available for BC therapy and are approved by the FDA in the USA [2], but adverse effects and the problematic development of drug resistance make it necessary to explore novel strategies and new drug targets.

An exciting new approach is to study cancer cells in microgravity (µ*g*), either with real (r-) µ*g* in space or simulated (s-) µ*g* by National Aeronautics and Space Administration (NASA), and European Space Agency (ESA)-acknowledged ground-based devices such as the random positioning machine (RPM) [3]. Cancer research conducted on the International Space Station (ISS) using the ISS National Laboratory includes, among others, clinical-grade stem cell investigations for future therapy, crystallization studies of proteins for improved drug discovery and delivery, and 3D cell culturing techniques. Spaceflights are rare and costly. Therefore, technologies to simulate microgravity are currently a hot topic in the field of space medicine and gravitational biology. Cancer research in space has become an important new research area. Devices to simulate microgravity are a new technology used to improve the current understanding of cancer biology [3].

The µ*g* environment offers special conditions that have the ability to change molecular mechanisms and cell signaling, thus controlling biological cellular processes [3,4]. For more than 30 years, it has been known that the µ*g* environment induces in vitro 3D tumor models (multicellular spheroids and organoids), which facilitate the study of the processes of cancer progression and metastasis on a molecular level in vitro [5].

For approximately four decades, researchers have focused on the impact of µ*g* on human benign and malignant cells. It is known that µ*g* induces various changes in humans, plants, microorganisms, animals and human cells [6,7,8,9,10].

Both r-µ*g* and s-µ*g* induce 3D growth in a multitude of benign and malignant cells, including MCF-7 and MDA-MB-231 [11,12,13,14]. Several research teams have already addressed the short-term effects of µ*g* on breast cancer cells, with the results published in [12,13,14,15,16,17,18,19]. These authors demonstrated the following findings: r-µ*g* and s-µ*g* induce changes in the cytoskeleton, extracellular matrix, focal adhesion, migration, proliferation, survival, apoptosis and growth in breast cancer cells. In particular, s-µ*g* alters the organization of the cytoskeleton [12,13], affecting proliferation, cell migration, apoptosis and cell cycle regulation [16,19]. A number of cancerous cell lines have already shown the capability to form multicellular spheroids (MCSs) without the use of any scaffolds in both r-µ*g* and s-µ*g* [20,21,22,23].

**Figure 1:**
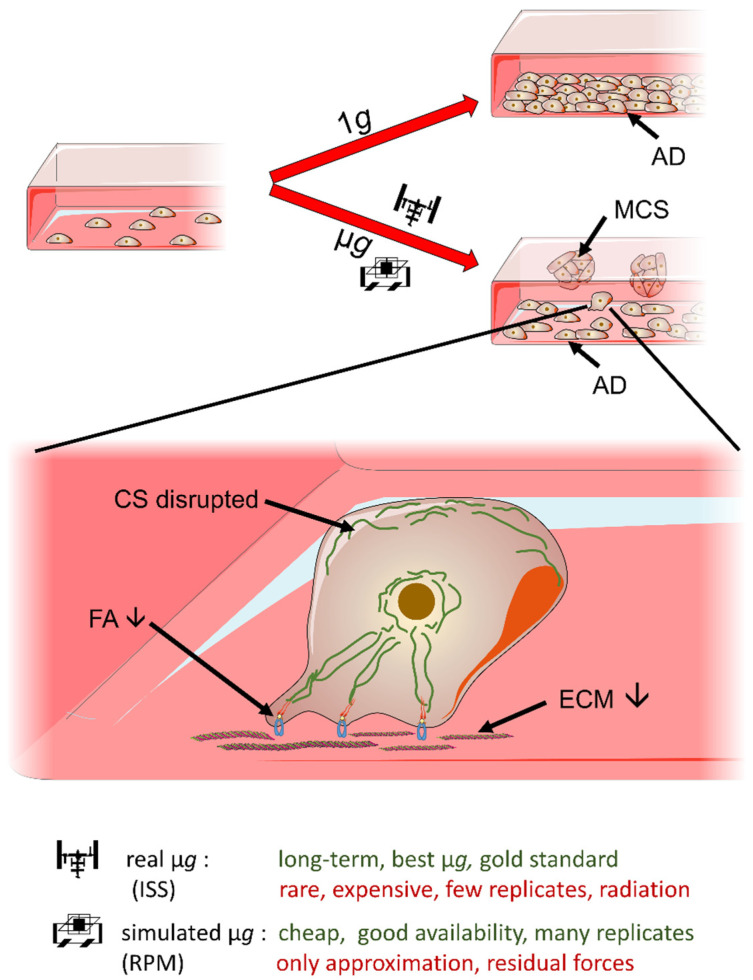
Breast cancer cells in microgravity. Schematic overview of effects, techniques and limitations. 1 *g*; normal gravity; µ*g*: microgravity; AD: adherent cells; MCS: multicellular spheroids; CS: cytoskeleton; FA: focal adhesion; ECM: extracellular matrix; ISS: International Space Station; RPM: Random Positioning Machine. Some elements of this figure were taken from the smart Servier Medical Art library (https://smart.servier.com/, accessed on 19 December 2022) published under the creative commons Attribution 3.0 Unported (CC BY 3.0) license (https://creativecommons.org/licenses/by/3.0/, accessed on 19 December 2022).

In earlier studies, we showed that MCF-7 and MDA-MB-231 breast cancer cells (BCCs) exposed to the RPM grew in the form of two phenotypes: one part continued growing as an adherent monolayer on the bottom of the cell culture flask, while the other part assembled within 24 h to three-dimensional aggregates, so-called spheroids (Figure 1) [13,14]. This finding was supported by others [12,18]. Interestingly, a recent study showed that benign MCF10 breast cells did not form spheroids after exposure to the RPM. Monti et al. [18] investigated normal (MCF10A) and cancerous (MCF-7) breast cells. The MCF10A breast cells split into two distinct morphological phenotypes, recognizable for the features of size and shape (large and small cells on the RPM), growing adherently to the substrate. They did not form spheroids, in contrast to the MCF-7 BCC [18].

However, the number of studies focusing on the underlying mechanisms and correlations of gene variation, interaction of drugs, differentiation and signaling pathways in this 3D spheroid environment is still very limited. Therefore, it is important to analyze 3D spheroids and compare their gene expression patterns with a patient-derived tumor cell gene expression. This knowledge can help us to evaluate 3D spheroids as a proxy for tissue from cancer patients.

In this study, MCF-7 BCCs, a luminal type, and MDA-MB-231 BCCs, a basal-like triple-negative type, were exposed to s-µ*g* for two weeks to observe changes during MCS formation.

The principal aims of the current study were: first, to study the impact of long-term s-µ*g* on breast cancer cells; second, to characterize the multicellular spheroid (MCS) model; and third, to improve understanding of the MCS model in the context of breast cancer patients. Therefore, a weighted gene co-expression network analysis (WGCNA) was conducted to derive the expression profiles of 35 breast cell lines from the HMS LINCS Database.

The selected and qPCR-verified genes were searched in the mammalian phenotype database and the human genome-wide association studies (GWAS) Catalog. The genes that had been up- or down-regulated in 3D spheroids in comparison with adherent cells in 1 *g* static controls and RPM monolayers were summarized. Data from breast tumor biopsies were downloaded from the HMS LINCS Database and differential gene expression data were evaluated by applying a weighted correlation network analysis (WGCN) [24] package for the co-expression network analysis, which is a widely used method not only for constructing gene networks, but also for detecting gene modules and identifying the central players, the so-called hub genes, within modules.

With the help of these novel bioinformatic studies, we found a positive association between the real metastatic microtumor environment and MCSs with respect to the extracellular matrix, cytoskeleton, morphology, different cellular signaling pathway key proteins and several other components.

## 2. Results

### 2.1. Cell Growth and Morphology

**Figure 2:**
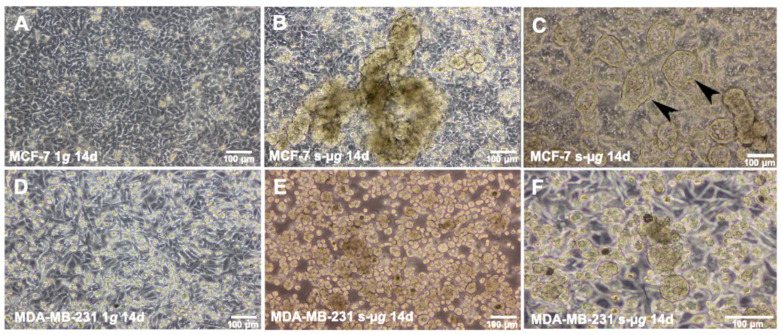
Morphology of RPM-exposed cells and characterization of 3D growth patterns: phase contrast microscopy of MCF-7 and MDA-MB-231 cells. (**A**–**C**): MCF-7 samples; (**A**): l *g* controls; (**B**,**C**): under RPM exposure, the formation of solid spheroids and ring-shaped glandular-like structures was observed. Both exhibited a large variation in size. (**D**–**F**): MDA-MB-231 samples; (**D**): l *g* controls; (**E**,**F**): numerous small, suspended MCSs were observed, along with a relatively small population of adherent cells. Black arrowheads indicate ring-shaped structures. Scale bars are 100 µm.

A total of 60 culture flasks containing 70% sub-confluent MCF-7 or MDA-MB-231 BCCs (*n* = 30 each group) were randomly assigned to the 14-day RPM experiments and to static 1 *g* controls. After 14 d, the 1 *g* control samples of both cell lines were 100% confluent and overgrown (Figure 2A,D). After 14 d, the RPM samples revealed two distinct phenotypes with different growth patterns. One part of the BCC grew adherently (AD) on the culture flask substrate comparable to the 1 *g* controls (Figure 1 and Figure 2B,E), while the other part grew three-dimensionally as MCSs (Figure 1 and Figure 2B,E). Interestingly, the MCF-7 cells exhibited glandular-like structures with a cellular morphology resembling epithelial polarity (Figure 2C). This phenomenon was also seen in earlier studies after a five-day RPM exposure of MCF-7 BCCs [6].

**Figure 3:**
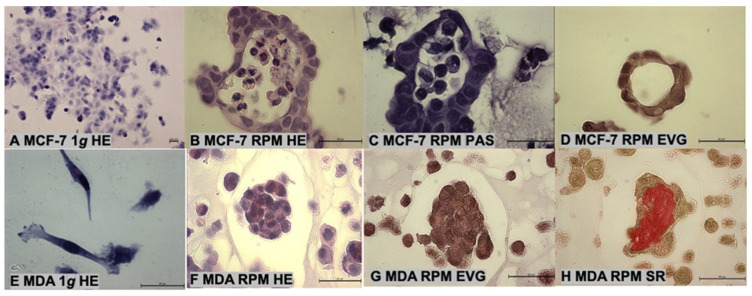
Morphology of multicellular spheroids. (**A**–**D**): MCF-7 samples; (**A**): hematoxylin eosin (HE) staining of l *g* control cells; (**B**): HE staining of a ring-shaped MCS; (**C**): periodic acid–Schiff (PAS) staining of a spheroid; (**D**): elastica van Gieson (EVG) staining of a small ring-shaped spheroid; (**E**–**H**): MDA-MB-231 (MDA) samples; (**E**): HE staining of l *g* control cells; (**F**): HE staining of a compact MCS; (**G**): EVG staining of spheroid; (**H**): sirius red (SR) staining showing collagen fibers in a spheroid. Scale bars are 30 µm.

The MCF-7 cell line exhibits an epithelial-like morphology. In addition, adherent cells create dome-like structures due to fluid accumulation between the culture flask bottom and cell monolayer, whereas the MDA-MB-231 BCCs show a spindle-shaped cell type (Figure 3A,E).

Hematoxylin eosin (HE), periodic acid–Schiff (PAS) and elastica van Gieson (EVG) staining demonstrated glandular structures in 14-day-old MCSs of MCF-7 BCCs (Figure 3B–D). In contrast, MDA-MB-231 spheroids revealed a compact structure (Figure 3F–H). Sirius red (SR) staining revealed an accumulation of collagen in MCSs (Figure 3H).

### 2.2. Expression Changes in Genes Related to the Cytoskeleton, Extracellular Matrix, Focal Adhesion Genes, Growth Factors and Signal Transduction

**Figure 4:**
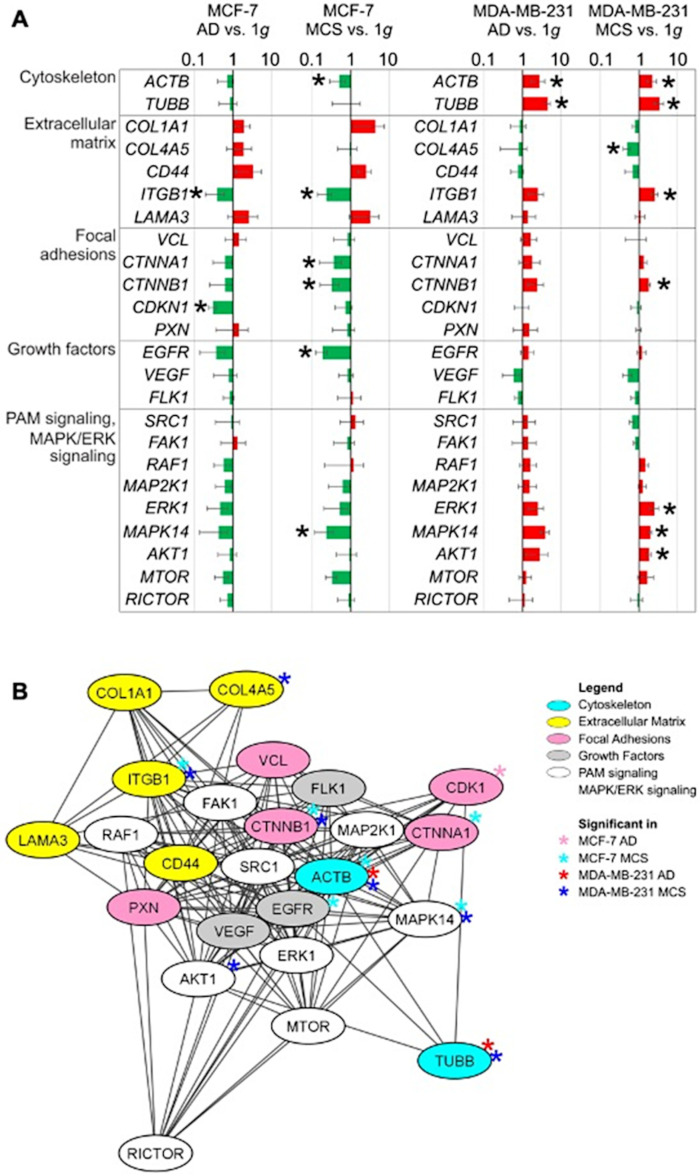
(**A**): qPCR fold changes of the studied genes in AD and MCS compared with 1 *g*. The red and green colors indicate up-regulated and down-regulated genes in the RPM-exposed samples, respectively. Significant regulations are indicated by a black asterisk (*p* < 0.05). (**B**): Functional interaction of genes analyzed in this study performed by STRING 11.5 (https://string-db.org/, accessed on 7 December 2022) and visualized by Cytoscape 3.8.2. The affiliation to functional gene groups is color-coded.

Figure 4A and Supplementary Figures S1–S5 display the qPCR results of this study and highlights our interpretation of the findings. A total of 24 genes of interest were investigated. These genes had shown their gravi-sensitivity and involvement in 3D growth and MCS formation in earlier studies [13,14,23]. A closer look revealed that the gene expression changed differently in both BCC types.

MCF-7 cells: The RPM-adherent cells, compared with the static 1 *g* controls (AD vs. 1 *g*), displayed no significant differential gene expression changes in ACTB, TUBB, COL1A1, COL4A5, CD44, ITGB1, LAMA3, VCL, CTNNA1, CTNNB1, PXN, EGFR, VEGF, FLK1, SRC1, FAK1, RAF1, MAP2K1, ERK1, MAPK14, AKT1, MTOR and RICTOR. The gene expressions of CDKN1 and ITGB1 were down-regulated in the AD cells compared with the 1 *g* control cells (Figure 4A and Figure S3).

MCSs, compared with the static 1 *g* controls (MCS vs. 1 *g*), showed significant down-regulations in the gene expressions of ACTB, ITGB1, CTNNA1, CTNNAb1, EGFR and MAPK14. No significant changes were measured for the following mRNAs: TUBB, COL1A1, COL4A5, CD44, LAMA3, VCL, CDKN1, PXN, VEGF, FLK1, SRC1, FAK1, RAF1, ERK1, AKT1, MAP2K1, MTOR and RICTOR.

MDA-MB-231 cells: A significant up-regulation of the ACTB and TUBB mRNA was measured in the RPM-adherent cells compared with the static 1 *g* controls (AD vs. 1 *g*). The other 22 genes of interest were not differentially displayed in the AD vs. 1 *g* group (Figure 4A and Figure S1–S5).

The COL4A5 gene expression was significantly down-regulated in MCSs compared with the static 1 *g* controls (MCS vs. 1 *g*) (Figure 4A and Figure S2). Significant up-regulations were measured for ACTB, TUBB, ITGB1, CTNNB1, ERK1, AKT1 and MAPK14 in MCS vs. 1 *g* (Figure 4A, Figure S1–S3 and S5).

Taken together, the results of the 14-day MCF-7 cell samples showed that most analyzed members of the MAPK/ERK signaling pathway were decreased, whereas these genes were up-regulated in MDA-MB-231 BCCs. Furthermore, the ITGB1 mRNA was significantly down-regulated in MCF-7 MCSs, whereas it was up-regulated in MDA-MB-231 cells.

Moreover, CTNNA1 and CTNNB1 were both significantly down-regulated in MCF-7 MCSs compared with 1 *g*, while CTNNB1 was up-regulated in MDA-MB-231 MCSs. The 24 genes analyzed by qPCR were further examined with regard to their possible interactions and mutual expression dependence.

A STRING/EMBL (European Molecular Biology Laboratory) analysis of these items represented in molecule action mode is shown in Figure 4B. Interactions of the altered focal adhesion molecules (CTNNA1, CTNNB1 and CDK1) with the regulated cytoskeleton protein ACTB were observed. In addition, the results indicate several interactions for ITGB1, CTNNB1, CDH1 and factors of MAPK/ERK signaling and the PAM pathway.

Moreover, we focused on the phosphorylation of proteins playing a central role in the signal transduction of spheroid formation. Phosphorylation reactions play an important role in the regulation of protein activities.

**Figure 5:**
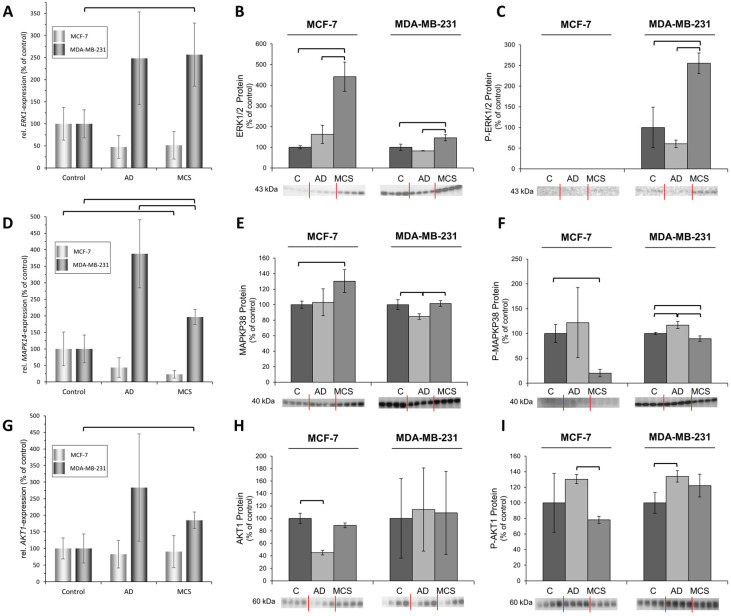
Quantitative real-time PCR and Western Blot analyses of both unphosphorylated and phosphorylated ERK1/2 (**A**–**C**), MAPK14 (**D**–**F**), and AKT1 (**G**–**I**) in MCF-7 and MDA-MB-231 cells exposed to 14 d of s-µ*g* on the RPM. *n* = 5 for qPCR, *n* = 4 for Western Blot. C: 1 *g* controls; AD adherent cells on the RPM; MCS: multicellular spheroids on the RPM; brackets indicate statistically significant differences at *p* < 0.05.

MCF-7 cells: The gene expressions of *ERK1, MAPK14* and *AKT1* were not changed in RPM-adherent cells as compared with static 1 *g* controls (AD vs. 1 *g*) (Figure 5A,D,G). A similar result was found for the gene regulation of *ERK1* and *AKT1* in MCS compared to 1 *g* control cells. In contrast, the *MAPK14* mRNA was significantly downregulated in MCS (Figure 5).

A counterregulatory effect was measured for the ERK1/2 protein synthesis, which was enhanced in MCS vs. 1 *g* and AD. No phosphorylated ERK1/2 protein was measured (Figure 5C). Moreover, MAPK14 protein was elevated in MCS compared to 1 *g,* whereas the phosphorylation process was reduced (Figure 5F). No significant change in the AKT1 protein synthesis in MCS vs. 1 *g* was found. Interestingly, the phosphorylation of AKT1 was increased in AD cells vs. MCS (Figure 5I).

MDA-MB-231 cells: The *ERK1*, *MAPK14* and *AKT1* mRNAs were up-regulated in MCS vs. 1 *g* (Figure 5A,D,G). The ERK1/2 unphosphorylated and phosphorylated proteins were elevated in MCS vs. 1 *g* and AD (Figure 5B,C). MAPK14 protein synthesis was unaltered in MCS, but p-MAPK14 protein was reduced compared to 1 *g* (Figure 5E,F).

The AKT1 protein synthesis was not altered after a 14-day RPM exposure, but the process of phosphorylation was increased in AD cells (Figure 5I).

### 2.3. Mammalian Phenotype Database and Human GWAS Catalogue

The above results showed that the key elements of the cytoskeleton, extracellular matrix and transmembrane receptor proteins respond differently to the RPM condition for the MCF-7 and MDA-MB-231 cell lines. The data confirmed the differential 3D growth patterns of these two BCC lines under RPM conditions. Furthermore, these findings raised the question of whether any of the tested genes or the co-expressed genes with these genes have an effect on BC progression and metastasis.

First, we checked the tested genes in the mammalian phenotype database. From there, we found several genes that could be targets (Supplementary Table S1). Some mouse mutation lines in *ACTB* showed the phenotype “increased mammary adenocarcinoma incidence” and “abnormal smooth muscle morphology”. Some mouse mutation lines in *AKT1* showed the phenotype “increased cell proliferation” and “mammary gland hyperplasia”. *CD44* has terms such as “increased circulating tumor necrosis factor level”, “decreased circulating tumor necrosis factor level”, “increased tumor necrosis factor secretion” and “abnormal cell physiology”. *CDKN1* and *COL1A1* have terms including “increased tumor incidence”. *CTNNA1* shows terms such as “abnormal embryonic tissue morphology” and “abnormal mammary gland epithelium physiology”. *CTNNB1* reveals the term “decreased cell proliferation”. EGFR exhibits the term “abnormal mammary gland morphology”. *FAK1* has the term “decreased tumor growth/size”. *MAPK14* shows the term “abnormal tumor incidence”.

Second, we queried the gene list against the GWAS Catalog (Supplementary Table S2) [25]. The results showed that several genes are associated with cancer incidence: *AKT1* is connected with endometrial cancer [26]; *CD44* is associated with progression-free survival in serous epithelial ovarian cancer treated with carboplatin and paclitaxel [27]; *CDH1* shows an association with colorectal cancer, as reported in two studies [28,29]; *COL1A1* is related to BC [30]; *COL4A5* is associated with non-melanoma skin cancer [31]; *EGFR* reveals an connection with BC [32]; *LAMA3* is associated with ovarian carcinoma [33]; and *TUBB* is linked to cervical carcinoma [34].

### 2.4. Weighted Gene Co-Expression Network Analysis

**Figure 6:**
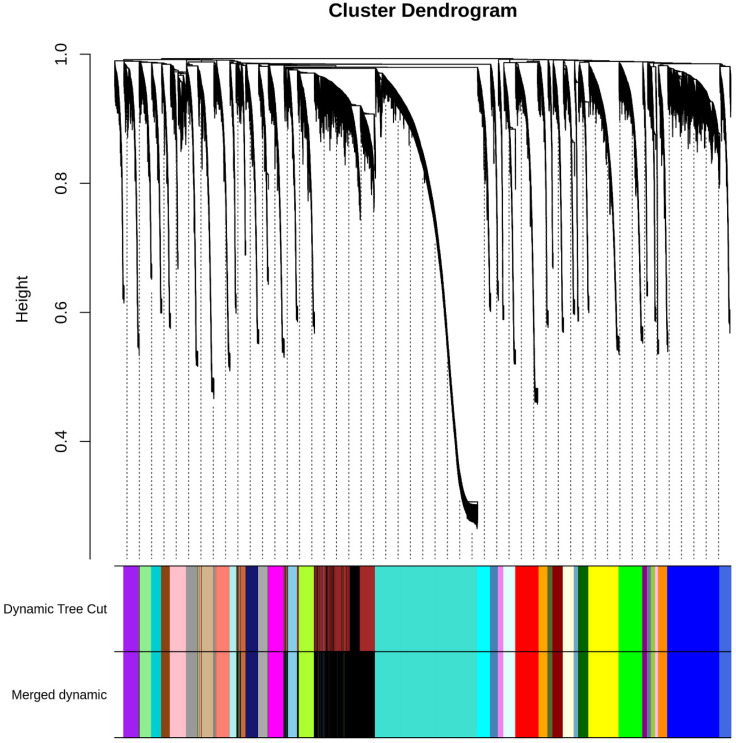
Clustering dendrogram of genes showing dissimilarity based on topological overlap, together with assigned merged module colors and the original module colors. The *y*-axis represents the network distance, as determined by topological overlap. Color blocks below the *x*-axis denote modules that genes were assigned to in each region. The upper panel shows the original clusters and the lower panel gives the clusters after the merging of modules, with a correlation of 0.7.

Furthermore, we performed weighted gene co-expression network analysis using the expression profile data of 35 breast cell lines from the HMS LINCS Database. The WGCNA [24] identified 38 modules (Figure 6, Supplementary Figures S6–S8) with 29,191 genes. From the above-verified genes, 18 remained (24 genes in total) classified. Moreover, 7 genes from these 18 belong to the same module, the blue module (Figure 6).

**Figure 7:**
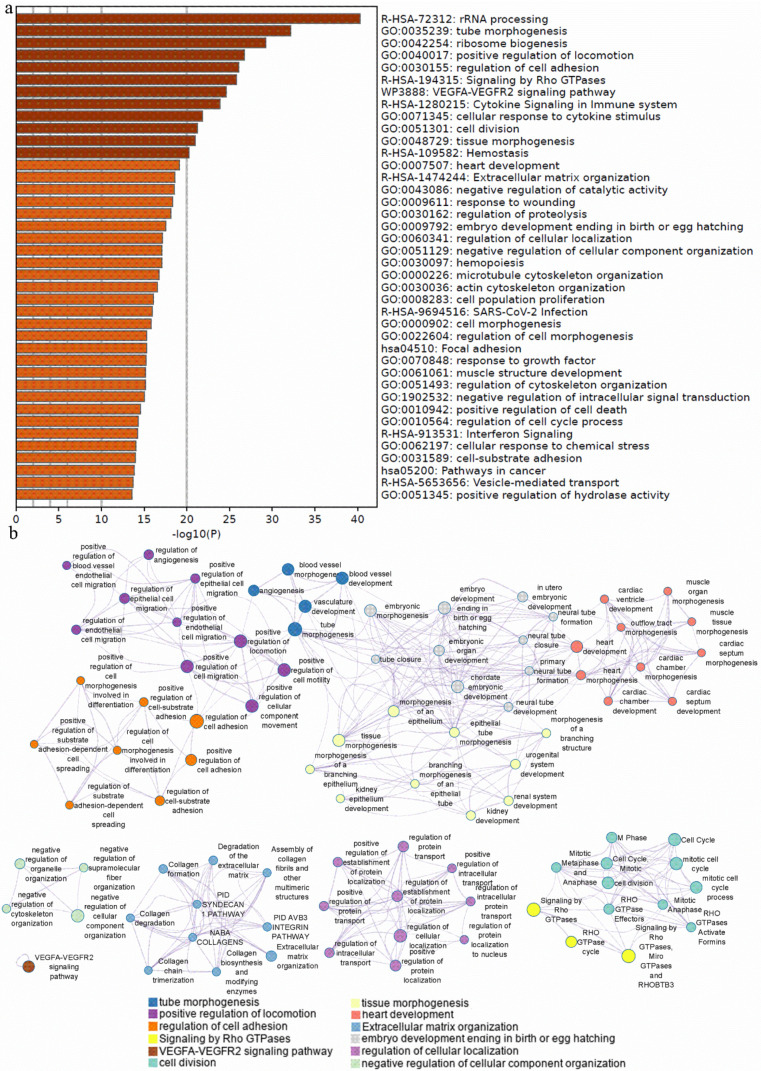
Pathway and process enrichment analysis: (**a**) Bar graph of top 40 enriched terms across input gene lists. The *x*-axis shows the significant level (−log10(*p* value)) by color, and the *y*-axis gives the enriched Gene Ontology, Reactome Pathway terms and Kyoto Encyclopedia of Genes and Genome terms; (**b**) the network of selected enriched gene annotation terms related to the current study is colored by cluster ID, where nodes sharing the same cluster ID are typically close to each other. The size of the circles shows the size of the gene in each term.

The annotation enrichment analysis (Figure 7a; Supplementary Table S3) demonstrated that the 19th enriched annotation term is “cell division” and the 11th term is “tissue morphogenesis”. These results showed that the blue modules did include genes with the function controlling cell morphology. For the KEGG pathway terms, “Proteoglycans in cancer” (hsa05205, Figure 7a and Supplementary Table S3) and “Pathways in cancer” (hsa05200, Supplementary Table S3) were the second and third most enriched KEGG pathway terms, ranking 38 and 44 (Supplementary Figure S9) for all annotations, respectively. The genes of these two pathways might be the target genes involved in cancer formation, progression and metastasis. To investigate the relationship between the enriched terms (including GO, KEGG, WikiPathways and Reactome), we selected the 20 most enriched terms to perform a network plot (Supplementary Figure S10 and Supplementary Table S3). Figure 7b shows the details of 12 term clusters related to cell/tissue morphology and cancer, or connected to these terms. The terms with a similarity of 0.3 are connected by edges. The plot indicates that the terms belonging to the cluster of the regulation of cell adhesion, positive regulation of locomotion, regulation of cell adhesion, tube morphogenesis, embryo development ending in birth or egg hatching, tube morphogenesis, tissue morphogenesis and heart development shared many genes from the blue module. These terms and genes are highly involved in tissue development. As we know, the WGCNA clusters of these genes in the same module (blue module) showed these genes co-expressed across BCC lines.

Taken together, these results verified our previous results indicating that these genes regulate the morphology of the two BCC types, and also showed that the WGCNA clustered a similar pattern of genes together. Moreover, we also can see that the term clusters of cell division and signaling by Rho GTPases found a network, while the negative regulation of cellular component organization, the regulation of cellular localization and the extracellular matrix organization clusters formed separate networks. Furthermore, 12 of the 20 most enriched clusters responded to tissue development and tissue morphology.

**Figure 8:**
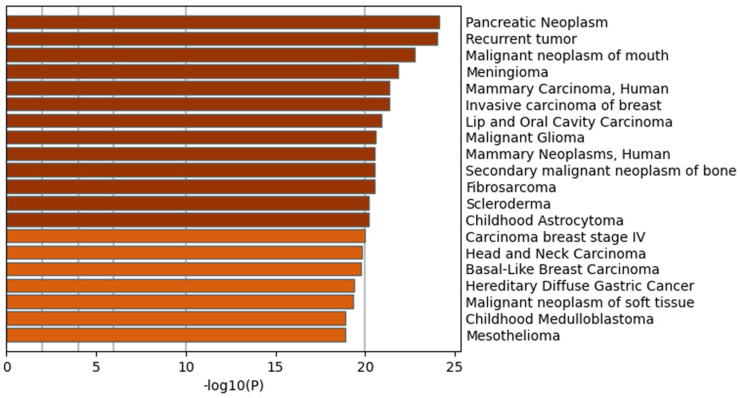
Enrichment of genes from the blue module in the human diseases database, DisGeNET. The −log10(*p* value) on the *x*-axis shows the significant level, which is also shown by colors. The *y*-axis indicates the enriched DisGeNET terms.

DisGeNET [35] is a platform for human disease-associated genes and variants. The enrichment analysis of the blue module was able to reveal whether the genes are associated with cancers, especially breast cancer. In Figure 8, it can be seen that the gene list is enriched for several terms related to cancer as well as breast function.

This indicates that the genes in the blue module are highly relevant for breast cancer. Combined with the above enrichment analysis (Figure 8), we propose that the genes in the blue module are associated with the 3D growth patterns of the two BCC lines.

## 3. Discussion

### 3.1. Long-Term RPM-Exposed BCCs Exhibit Alterations in Growth, Cytoskeleton and Extracellular Matrix

The 3D spheroid formation is a complex multistep process involving cell detachment, cell migration and the reorganization of the cytoskeleton into a rigid extracellular matrix to shape the solid spheroids and ring-shaped glandular-like structures [5]. After a 14-day RPM exposure, both BCC lines revealed MCS and an adherent monolayer. The MDA-MB-231 cells showed compact 3D MCSs, while the MCF-7 BCCs revealed duct-like MCSs exhibiting a lumen together with compact spheroids (Figure 1). These duct-like MCSs had also been observed after a five-day RPM experiment with MCF-7 cells [13], while after 24 h only, compact MCSs were detectable [13,14].

Our findings are also in concert with data published by others [12]. The question remains as to whether these effects might be triggered by shear forces introduced by the movement of the RPM. While the flasks were completely filled with medium to reduce the occurrence of these forces, they cannot be completely avoided, especially in those flasks positioned slightly outside the rotational center of the frames. While it is difficult to measure the shear forces directly, some groups have aimed to analyze them indirectly, using either biosensors or numeric simulations [36,37]. The latter study found that adherent cells are exposed to a maximum shear stress of 100 mPa at RPM rotation velocities of 60°/s. Interestingly, a recent work studied the shear forces necessary to detach MCF-7 cells, among others, from their substrate [38]. It was shown that, to detach 50% of the cells, shear stresses of approximately 4.5 Pa were necessary, a value 40 to 50 times higher than those occurring during RPM exposure. Furthermore, experiments in real µ*g* in space, in an environment devoid of any shear forces, also yielded MCSs from originally attached cells, indicating that s-µ*g*-induced MCS formation is not a mechanical stress-related artifact [39,40]. We therefore assume that shear forces are not among the major factors inducing the observed effect, and should not have a major confounding influence on the performed gene expression analyses.

It is well-known that microgravity alters the structure of the BCC cytoskeleton within a very short time [41]. The cytoskeleton is a complex framework of biopolymers composed of different filamentous components—actin, microtubules and intermediate filaments—linked to themselves and each other to form a structurally connected network [42]. *ACTB* gene expression was significantly reduced in MCF-7 MCSs, whereas *ACTB* was up-regulated in the ADs and MCSs of MDA-MB-231 BCCs, which might be explained by the different characteristics of the two BCC lines. Previous studies have shown that *ACTB* deregulation was usually detected in tumors and affected the polymerization of *ACTB* at the leading edge in tumor cells, resulting in tumor progression and metastasis [43].

Additionally, the *TUBB* mRNA was unaltered in MCF-7 cells, whereas it was up-regulated in the ADs and MCSs of MDA-MB-231 cells (Figure 4A). Microtubules composed of polymerized tubulin (*TUBB* gene) are the major load-distributing parts of the cell. They are important for chromosome separation during mitosis, and for intracellular transportation of vesicles and organelles. The microtubule network has been shown to be sensitive to µ*g* [15,44]. Other studies have shown that the overexpression was significantly related to distant metastases of BC [45].

Other interesting findings of this study were alterations in the extracellular matrix (ECM) components, which are major elements of the tumor microenvironment. *COL1A1* was not changed after the RPM exposure of both cell types. The *COL4A5* mRNA was down-regulated in MDA-MB-231 MCSs (Figure 4A). In vitro studies demonstrated that the expression of *COL4A5*/*COL4A6* genes was down-regulated in colon cancer cell lines, suggesting that normal basement membranes were disrupted in progressive tumors, accompanied by a down-regulation of gene expression [46].

The remodeling of integrin-beta plays an important role in cancer development and progression. The up-regulation of *ITGB1* in MDA-MB-231 MCSs and its down-regulation in MCF-7 MCSs indicated the different malignancies of the two BCC lines [47]. Elevated *ITGB1* levels have been linked to lower survival. The silencing of *ITGB1* inhibited TNBC cell migration and invasion [48].

### 3.2. Simulated Microgravity Influences the Expression of Focal Adhesion Factors, Growth Factors and Signal Transduction

The *CTNNA1* and *CTNNB1* mRNAs were both down-regulated in RPM-engineered MCF-7 MCSs, while *CTNNB1* was up-regulated in MDA-MB-231 MCSs. In contrast, after 24 h of RPM exposure for MCF-7 cells, both the *CTNNA1* and *CTNNB1* mRNAs were not differentially displayed in MCF-7 cells [14]. Within 24 h of RPM exposure, β-catenin protein was located in the nucleus and cytoplasm of adherent 1 *g* and RPM-AD MCF-7 cells [14]. In MCSs, β-catenin was detectable in the membranes. These data suggest its role in cell detachment and MCS formation. β-catenin overexpression seems to be involved in the process of differentiation. In vivo, using the hind-limb unloading mouse model, it was shown that β-catenin could, in part, attenuate the osteoblast differentiation reduction induced by s-µ*g* [49]. In addition, β-catenin is involved in TNBC development [50].

We focused on epidermal growth factor receptor (EGFR), which is known to predict a poor outcome in BC and is associated with proliferative effects and progression [51,52]. The EGFR acts via MAPK and PI3K/Akt pathways. Interestingly, long-term µ*g* did not alter the gene expression of *EGFR* in the MDA-MB-231 cells (Figure 4A), while *EGFR* was significantly down-regulated in the MCSs of the MCF-7 cell line. A similar result for *EGFR* was found when human colorectal carcinoma cells (MIP-101) were studied under conditions of µ*g* [53]. The *EGFR* gene expression was stable in MDA-MB-231 cells, which might explain why these highly malignant BCCs develop a less malignant phenotype under µ*g* conditions, a finding reported by others [54,55]. This result is supported by data of rat osteoblasts showing that the mRNA levels for the EGF receptor were not altered by r-µ*g* during a spaceflight [56]. An increase in *EGFR* in MCSs was measured after a 24 h RPM exposure of PC-3 prostate cancer cells [23], indicating its early involvement in MCS formation.

In addition, *VEGF* levels were not changed after the 14-day RPM exposure compared with the static 1 *g* controls. In addition, *VEGFR2* (*FLK1*) was also not altered in either BCC line. In contrast, both genes were significantly down-regulated in MCF-7 MCSs after a 5-day s-µ*g* exposure [13]. This suggests that the signaling of VEGF and VEGFR2 is not the dominant mechanism when MCF-7 cells react to µ*g*.

The EGFR acts via downstream pro-oncogenic signaling pathways, including the RAS-RAF-MEK-ERK MAPK and AKT-PI3K-mTOR pathways [57]. The binding of VEGF to VEGFR activates the MAPK and AKT1 pathways, which are involved in proliferation and survival [58]. In addition, *ERK1* was up-regulated in MDA-MB-231 MCSs after 14 days in s-µ*g*, while it remained unaltered in MCF-7 cells. *MAPK14* was up-regulated in MDA-MB-231 MCSs and down-regulated in MCF-7 MCSs. *AKT1* was elevated in MDA-MB-231 MCSs, indicating the potential for TNBC to proliferate and for increased survival.

### 3.3. Mammalian Phenotype Database and Human GWAS Catalog

Advances in molecular biology have provided evidence of gene functions. However, from the point of view of the phenotype, many genes show pleiotropy, making the interpretation of the link between genes and the phenotype complicated. To help with this, the human GWAS Catalog database [25] and the mammalian phenotype database [59] provide useful information to link the genes with the phenotype. The human GWAS Catalog database collects genome-wide associated significant SNPs and the candidate genes of a wide range of phenotypes. Therefore, we queried the human GWAS Catalog regarding the genes we selected for qPCR. The results showed eight genes associated with phenotypes related to cancer. The mammalian phenotype database collects mouse mutation lines with the phenotype. The investigation of the gene list on the mammalian phenotype database revealed nine genes related to tissue morphology or cancer. In total, we found supportive evidence for 13 genes.

### 3.4. Interaction Network of Selected Genes Evaluated by STRING Analysis and Cytoscape

Cytoscape analysis showed several interactions between the investigated factors, with the majority of them exhibiting a large number of interactions. Among others, *ACTB*, *EGFR*, *CTNNB1*, *MAP2K1*, *AKT1*, *ERK1*, *MTOR* and *ITGB1* are indicated as being dominant target genes, as many arrows point to their icons (Figure 4B). The VEGF and EGFR pathways are involved in biological processes such as tumor growth and progression, angiogenesis and metastasis. In many cancer types, they are targeted by multi-kinase anticancer therapy [60,61].

There are interactions between *COL1A1, ACTB* and *CTNNB1.* Collagen type I induces cytoskeletal alterations, migration and proliferation, indicating its involvement in promoting gastric cancer invasion and metastasis [62]. Moreover, it was demonstrated that collagen type I is involved in the beta-catenin tyrosine phosphorylation and the translocation to the nucleus [62]. EGF stimulation induces the translocation of β-catenin into the nucleus, and thus increases its transactivation without altering its stability and phosphorylation by GSK-3β [63]. FTC-133 cells cultured in s-µ*g* revealed two different phenotypes: one part grew adherently and the other part formed MCSs. Dexamethasone application inhibited spheroid formation dose-dependently [64]. Downstream of the cadherin complex, *CTNNB1* was not influenced significantly by DEX. However, β-catenin was translocated from the plasma membrane into the nucleus in the presence of dexamethasone, suggesting the involvement of the Wnt/β-catenin pathway [64].

Ser552 phosphorylation of β-catenin by AKT led to its dissociation from cell–cell contacts, accompanied by enhanced transcriptional activity and tumor invasion [65]. In addition, AKT is involved in controlling the cellular metabolism, survival and growth of BCCs [66]. AKT is the key factor of the PI3K/AKT/mTOR (PAM) signaling pathway. The deregulation of the PAM pathway is common in BC and prostate cancer [22]. Human promyelocytic leukemic HL-60 cells cultured under s-µ*g* created by a rotary cell culture system (RCCS) exhibited reduced cell proliferation and viability. These effects were accompanied by a decreased expression of PCNA and phosphorylated ERK1/2 and AKT proteins [67]. Taken together, s-µ*g* inhibited mitogenic and survival pathways through ERK1/2 and AKT signaling in HL-60 cells [67], which is comparable to the results obtained from the MCF-7 cells (Figure 4A). In contrast, triple-negative BCCs, such as the MDA-MB-231 cells, revealed a different reaction when they were exposed to s-µ*g* for 14 d, indicating that the ERK1 and AKT signaling processes were activated in these cells (Figure 4A and Figure 5C,I).

*ITGB1* gene expression was clearly different in both cell lines. *ITGB1* was significantly down-regulated in the MCSs of MCF-7 cells compared with 1 *g*, whereas the *ITGB1* mRNA was up-regulated in MDA-MB-231 MCSs. After a 24 h and five-day RPM exposure of MCF-7 cells, *ITGB1* was significantly down-regulated in MCSs [13]. Integrins are membrane receptors, and they play an important role in cell adhesion and, among others, metastasis. Integrins link the actin cytoskeleton with the ECM and are involved in mechanotransduction [68]. Beta-integrins are primarily responsible for targeting the sites of focal adhesions. A loss of integrin and/or vinculin may reduce their function. Recently, we investigated MDA-MB-231 cells for 24 h under s-µ*g* and measured the down-regulation of *ITGB1* in AD and MCS cultures [14]. After 14 d, *ITGB1* was highly elevated in MCSs (Figure 4A). Under long-term µ*g* exposure, cancer cells depend on adhesion to evade cell death. Other cell types such as FTC-133 thyroid cancer cells and mesenchymal stromal cells showed a reduced *ITGB1* gene expression when they were exposed to space or s-µ*g* conditions [64,69], whereas the *ITGB1* mRNA was up-regulated in chondrocytes [70]. In summary, we discovered that the reaction of *ITGB1* to environmental stresses such as µ*g* is cell-type-dependent and time-dependent.

Interestingly, the gene expression of *FAK1/PRK2* (FAK/protein tyrosine kinase 2 (PTK2)), which plays a role in mechanosensing and transduction, supports proliferation and migration, and links the ECM and the cytoskeleton, was not significantly changed in either cell line after 14 d. This result is comparable to the *FAK1/PTK2* expression data of both BCC cell lines grown under s-µ*g* for 24 h [14]. FAK is important for BC progression and metastasis. It has been shown that FAK depletion switches phosphotyrosine-containing proteins from focal adhesions to invadopodia through the regulation of c-Src activity [71].

In addition, we measured the expression of *PXN*. Paxillin is expressed at FA sites and supports the adherence of human cells to the ECM. FAK1 and vinculin can bind to paxillin. Long-term exposure to s-µ*g* did not alter the expression of *PXN* in either cell line. This is in contrast to the data of short-term studies. In MCF-7 cells, the *PXN* gene in s-µ*g*-exposed cells was significantly down-regulated compared with 1 *g*, while in MDA-MB-231 cells, the *PXN* mRNA was not altered [14]. *VCL1*, *PXN* and *FAK1* were not changed after 14 d of s-µ*g*, indicating the early activity of these genes in the metastasis process.

The *MAPK14* (p38α; mitogen-activated protein kinase 14) gene was differentially expressed in both cell lines. *MAPK14* was significantly up-regulated in MDA-MB-231 MCSs and down-regulated in MCF-7 MCSs compared with 1 *g* (Figure 4A). The p38α pathway plays a dual role in the organism. MAPK14 is a negatively regulating proliferation in normal cells and among others in BCCs [72]. In parallel, MAPK14 is involved in tumor-related processes such as cell metabolism, invasion, inflammation and angiogenesis [72].

One space experiment showed that human bone marrow-derived mesenchymal stem cells differentiated from osteogenesis to adipogenesis [73]. The authors demonstrated that r-µ*g* reduced FAK activity and derepressed AKT activity. An increase in p38 MAPK activity and the de-repression of AKT activity might induce differentiation to adipogenesis [73]. In addition, p38 MAPK signaling mediates the s-µ*g* (RCCS)-induced chondrogenesis of adipose-derived MSCs [74].

### 3.5. Weighted Gene Co-Expression Network Analysis

In addition to the protein–protein interactions, we conducted WGCNA with global gene expression levels from 35 BCC lines. As the genes we explored using qPCR are related to tissue morphology in two BCC lines, we aimed to see if these genes determine the 3D formation of breast cancer cell lines in a general way, and further, whether we can use the co-expression to find genes in the same pathway with the genes tested using qPCR. We found that the blue module harbored 7 (*ACTB*, *CD44*, *EGFR*, *ITGB1*, *PXN*, *TUBB* and *VCL*) out of 18 genes that were successfully analyzed using WCGNA. As a result, we further investigated the blue module using gene annotation enrichment analysis. The enrichment analysis showed that the blue module comprised the cluster of genes responding to the morphology of breast cancer.

Taken together, these results demonstrated an association between the real metastatic microtumor environment cells on the RPM with respect to several components, including the extracellular matrix, cytoskeleton, morphology and different cellular signaling pathway key proteins. The above results showed that the key elements (*ERK1*, *AKT1*, *MAPK14*, *EGFR*, *CTNNA1*, *CTNNB1*, *ITGB1*, *COL4A5*, *ACTB* and *TUBB*) respond differently to the RPM condition for the MCF-7 and MDA-MB-231 cell lines.

## 4. Materials and Methods

### 4.1. Cell Culture

MCF-7 and MDA-MB-231 human mammary adenocarcinoma cells (catalog numbers HTB-22 and HTB-26, respectively; American Type Culture Collection, Boras, Sweden) were the two cell lines used to study the effects of s-µ*g* on BCCs. The cell culture was performed according to protocols published earlier [13,14].

The cells were grown in RPMI 1640 medium (Thermo Fisher Scientific, Nærum, Denmark), supplemented with 10% fetal calf serum (FCS) (Sigma-Aldrich Chemie GmbH, Munich, Germany) and 1% penicillin/streptomycin (Thermo Fisher Scientific), and maintained under standard cell culture conditions at 37 °C and 5% CO_2_.

One day prior to the RPM experiment, 1 × 10^6^ cells were counted and seeded into T25 cm^2^ vented cell culture flasks (Sarstedt, Nümbrecht, Germany) and 2.5 × 10^5^ cells were seeded into the slide flasks (Thermo Fisher Scientific) for F-actin staining and immunohistochemical investigations. Each flask was completely filled with medium without air bubbles. The flasks were installed on the center plate of the RPM and run for 14 d (*n* = 15 flasks) using the real random mode. Then, 1 *g*-static controls were prepared in parallel (*n* = 15 each) and stored next to the device in the same incubator.

The medium was exchanged every 48 h and the cells were investigated by phase contrast microscopy. For the medium exchange, the flasks were placed upright for one minute to let any cells in the supernatant sediment reach the bottom. Next, 2/3 of the medium was carefully aspirated with a pipette without catching any MCS or cell material. The flasks were then refilled with fresh medium in a bubble-free manner and put back on the RPM. The cells and MCSs were harvested at day 14. The supernatant was collected and centrifuged at 4 °C to collect the MCSs. The MCSs were collected and stored in liquid nitrogen and one part was fixed in 4% paraformaldehyde (PFA). The method for cell harvesting was published in detail previously [14].

### 4.2. Simulated Microgravity Using the Desktop Random Positioning Machine

Microgravity was simulated using a desktop RPM (Airbus Defense and Space, former Dutch Space, Leiden, the Netherlands). The method for s-µ*g* exposure was published in [14,75].

For each experiment, 15 T25 cm^2^ cell culture flasks (Sarstedt) were placed near the center of rotation to limit residual accelerations and centrifugal effects.

The desktop RPM used was small enough to fit into a CO_2_ incubator under standard conditions of 5% CO_2_ and 37 °C. During the RPM experiment, 15 T25 cm^2^ cell culture flasks served as static l *g* control experiments, as described in [14].

### 4.3. Histochemical Staining

Hematoxylin and eosin and elastica van Gieson staining procedures were used to study the MCSs. Furthermore, periodic acid–Schiff (PAS) staining was applied to investigate the cellular basement membranes of the MCS cells. In addition, 3 µm slices of MCS were prepared and stained with an aqueous solution of picrosirius red (0.1%) to detect collagen accumulations [76,77,78].

### 4.4. Microscopy

Phase contrast and light microscopy procedures were published earlier in [14].

### 4.5. RNA Extraction and Quantitative Real-Time Polymerase Chain Reaction (qPCR)

The RNA isolation and qPCR analysis were conducted as in [14,79].

The primers used are listed in Table 1.

### 4.6. Sample Collection and Protein Extraction

After the 14-day experiment, the samples with the BCC were collected. The method was published earlier in Sahana et al. [14].

For protein extraction, the cell pellets were lysed with lysis buffer (RIPA + protease/phosphatase inhibitor; 1:100) by adding half the amount to pellet size, vortexed for 30 sec and kept on ice for 30 min. During that time, the tubes were vortexed every 10 min for 30 sec. Then followed a sonification procedure for 45 s using a Branson 2210 Ultrasonic bath (Buch & Holm, Herlev, Denmark) and the samples were centrifuged for 10 min at 4 °C and 13,000 rcf. Finally, the supernatants were transferred to new Eppendorf cups and the protein concentration was measured using an ELISA machine.

### 4.7. Western Blot Analysis

The protein samples were boiled at 99 °C for 5 min. The wells of the Criterion TGX Stain-Free Gel (Cat-#5678084, Bio-Rad) were rinsed with 200 µL of running buffer twice and the samples loaded. The molecular weight marker (Cat. #161-0373, Bio-Rad) was applied.

The gel ran for 30 min at 250 V with the running buffer (MOPS, Cat. #161-0788, Bio-Rad). After the run, the gel was removed from the plastic and exposed to UV light to obtain an image for total protein quantification and normalization. During the gel UV scan, the polyvinylidene difluoride membrane (Cat. #1620177, Bio-Rad) was activated with ethanol 99.9% for 5 min. Then, followed a washing step with transfer buffer.

The fiber pads and filter papers were soaked in transfer buffer for a few minutes before the transfer. The gel, the membranes and the fiber pads were stacked and put into the transfer machine for 30 min and 100 V. Afterwards, the membrane was read with UV light to determine the total protein transferred and washed for 10 min with Tris-buffered saline-Tween 20 (TBST) to remove excess transfer buffer.

Then, the membrane was incubated for 5 min with the Every-Blot blocking buffer (Bio-Rad). After the blocking procedure, the membrane was incubated with the primary antibody solution in blocking buffer for 2 h at room temperature (RT) or overnight at 4 °C. The antibodies used are listed in Table 2.

Then, the membrane was washed four times with TBST for 10–15 min. This washing step was followed by the incubation with the secondary antibody diluted in blocking buffer for 2 h at RT (Table 2). The same washing steps were carried out as after incubation with the primary antibody.

Finally, the detection was carried out using the Clarity Western ECL Substrate (Cat. #1705061, Bio-Rad) solution according to the manufacturer’s instructions. Both reagents were mixed at 1:1 and distributed on the membrane surface in the dark for 5 min.

The images were obtained and analyzed with Syngene Pxi imager and the Syngene Gene Tools analysis software 1.8.6. An overview of the raw data is given in Supplementary Figure S11.

### 4.8. Weighted Gene Co-Expression Network Analysis: HMS LINCS Database

The data for BC were download from the HMS LINCS Database with the ID: HMS_Dataset_20348 (http://lincs.hms.harvard.edu/db/datasets/20348/, accessed on 12 May 2022). The resulting counts table was normalized to reads per kilobase of transcripts per million mapped reads (RPKM). The value was read into the WGCNA package for co-expression network analysis [24]. The analysis was conducted according to the tutorials from WGCNA [24,80]. The samples were clustered to visualize the potential outlier (Supplementary Figure S1). As shown in Supplementary Figure S6, there was no clear outlier. Therefore, we included all samples. We chose the “signed hybrid” module type for analysis, and the soft threshold was set to three (Supplementary Figure S7) as the first value reached 0.8 of the scale-free fit index. The threshold for the size of modules was set to 30 and the modules merged with a correlation of 0.7 (Supplementary Figure S8).

### 4.9. Gene Annotation and Gene Enrichment Analysis

The qPCR-verified genes were used to search the human GWAS Catalog database [25] and mammalian phenotype database [59]. The same gene list was also used to determine the gene cluster with a similar expression pattern across different cancer cell types from the WGCNA result. We extracted genes in the blue module and performed enrichment analysis with Metascape [80].

### 4.10. STRING Analyses

The interactions between proteins were determined using the STRING v11.5 platform [81]. Pairwise protein connections were created under the medium confidence (interaction score > 0.4) constraint considering all seven available interaction sources. The network visualization was carried out using Cytoscape 3.8.2.

### 4.11. Statistical Analysis

The statistical analysis was performed using the GraphPad Prism 7.01 software (GraphPad Software, Inc., San Diego, CA, USA). The differences between the s-µ*g* samples and the related controls were assessed with the Mann–Whitney U-test, and *p*-values < 0.05 were considered significant.

## 5. Conclusions

**Figure 9:**
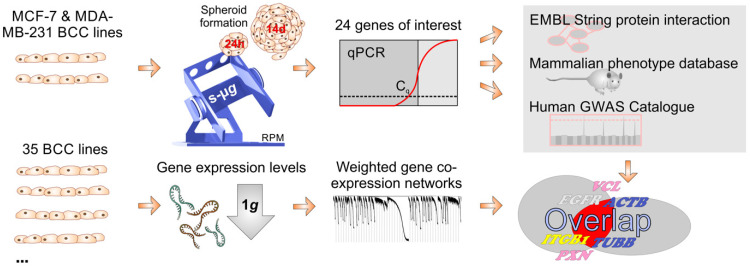
Graphical summary of the bioinformatics workflow. The s-µ*g*-related gene expression changes of 24 candidate genes were checked by qPCR depending on the aggregation state of the cells (adherent or spheroids). The genes were then checked for known protein–protein interactions and documented pathological relevance. In parallel, the gene expression of 35 BCC lines was evaluated in silico under 1 *g* conditions using a weighted gene co-expression network analysis. The results were subsequently pooled.

MCF-7 and MDA-MB-231 BCCs were investigated in a long-term s-µ*g* experiment. Over the 14-day RPM exposure, the cells grew into the form of two phenotypes: (1) as an adherent 2D monolayer and (2) as 3D multicellular spheroids (Figure 9). The *ERK1*, *AKT1*, *MAPK14*, *EGFR*, *CTNNA1*, *CTNNB1*, *ITGB1*, *COL4A5*, *ACTB* and *TUBB* gene expression of MCSs was differentially regulated in the MCF-7 and MDA-MB-231 cells. The bioinformatic analyses demonstrated the positive association between the real metastatic microtumor environment and MCSs with respect to the cytoskeleton, the extracellular matrix, focal adhesion and EGF/MAP signaling, dependent on the breast cancer type. Overall, this long-term study provides novel knowledge regarding spheroid formation and the growth behavior of BCCs in microgravity.

## Figures and Tables

**Table 1:** Primer sequences for qPCR.

Gene	Primer Name	Sequence
*18S rRNA*	18S-F	GGAGCCTGCGGCTTAATTT
	18S-R	CAACTAAGAACGGCCATGCA
*ACTB*	ACTB-F	TGCCGACAGGATGCAGAAG
	ACTB-R	GCCGATCCACACGGAGTACT
*AKT1*	AKT1-F	CTTCTATGGCGCTGAGATTGTG
	AKT1-R	CAGCATGAGGTTCTCCAGCTT
*CD44*	CD44-F	ACCCTCCCCTCATTCACCAT
	CD44-R	GTTGTACTACTAGGAGTTGCCTGGATT
*CDKN1*	CDKN1-F	GACCTGCAACCGACGATTCT
	CDKN1-R	GGGCGTCTGCTCCACAGA
*COL1A1*	COL1A1-F	CGATGGATTCCCGTTCGAGT
	COL1A1-R	GAGGCCTCGGTGGACATTAG
*COL4A5*	COL4A5-F	GGTACCTGTAACTACTATGCCAACTCCTA
	COL4A5-R	CGGCTAATTCGTGTCCTCAAG
*CTNNA1*	CTNNA1-F	AATTTAGCGCTCGCCCAG
	CTNNA1-R	ACAAGGGTTGTAACCTGTGTAA
*CTNNB1*	CTNNB1-F	GAAACAGCTCGTTGTACCGC
	CTNNB1-R	ATCCACTGGTGAACCAAGCA
*EGFR*	EGFR-F	TTGCCGCAAAGTGTGTAACG
	EGFR-R	GAGATCGCCACTGATGGAGG
*ERK1*	ERK1-F	ACCTGCGACCTTAAGATTTGTGA
	ERK1-R	AGCCACATACTCCGTCAGGAA
*FAK1*	FAK1-F	TGTGGGTAAACCAGATCCTGC
	FAK1-R	CTGAAGCTTGACACCCTCGT
*FLK1*	FLK1-F	TCTTCTGGCTACTTCTTGTCATCATC
	FLK1-R	GATGGACAAGTAGCCTGTCTTCAGT
*ITGB1*	ITGB1-F	GAAAACAGCGCATATCTGGAAATT
	ITGB1-R	CAGCCAATCAGTGATCCACAA
*LAMA3*	LAMA3-F	AAAGCAAGAAGTCAGTCCAGC
	LAMA3-R	TCCCATGAAGACCATCTCGG
*MAP2K1*	MAP2K1-F	CGTTACCCGGGTCCAAAATG
	MAP2K1-R	TCCAAGTTGGTCTCCGCA
*MAPK14*	MAPK14-F	TGTTTCCTGGTACAGACCATATT
	MAPK14-R	CATGGCTTGGCATCCTGTT
*MTOR*	MTOR-F	ATCTTGGCCATAGCTAGCCTC
	MTOR-R	ACAACTGGGTCATTGGAGGG
*PXN*	PXN-F	CATGGACGACCTCGACGC
	PXN-R	CAAGAACACAGGCCGTTTGG
*RAF1*	RAF1-F	GGGAGCTTGGAAGACGATCAG
	RAF1-R	ACACGGATAGTGTTGCTTGTC
*RICTOR*	RICTOR-F	GGAAGCCTGTTGATGGTGAT
	RICTOR-R	GGCAGCCTGTTTTATGGTGT
*SRC1*	SRC1-F	CCACCTTTGTGGCCCTCTAT
	SRC1-R	CCTCTGTGTTGTTGACAATCTGG
*TUBB*	TUBB-F	CTGGACCGCATCTCTGTGTACTAC
	TUBB-R	GACCTGAGCGAACAGAGTCCAT
*VCL*	VCL-F	GTCTCGGCTGCTCGTATCTT
	VCL-R	GTCCACCAGCCCTGTCATTT
*VEGF*	VEGF-F	CTACCTCCACCATGCCAAGTG
	VEGF-R	GCGCTGATAGACATCCATGAAC

All sequences are given in the 5′-3′ direction.

**Table 2:** List of antibodies used for Western blot analysis.

Antibody	Catalog nr. and Company	Dilution Primary Antibody	Host Species	Secondary Antibody	Dilution Secondary Antibody
AKT 1	Ab 89402, Abcam	1:1000	Mouse	#7076S, Cell signalling	1:4000
P-Akt1	# 92715, Cell signalling	1:1000	Rabbit	#7074S, Cell signalling	1: 4000
MAPKp38	MA5 15116, Thermo Fisher	1:1000	Mouse	#7076S, Cell signalling	1: 4000
P-MAPKp38	446845G, Invitrogen	1:1000	Rabbit	#7074S, Cell signalling	1: 4000
ERK1/2	#9102, Cell signalling	1:1000	Mouse	#7076S, Cell signalling	1: 5000
P-ERK1/2	#9101, Cell signalling	1:1000	Rabbit	#7074S, Cell signalling	1: 5000

## Data Availability

The datasets analyzed for this study can be provided by contacting the corresponding author.

## Supplementary Materials

The following supporting information can be downloaded at: https://www.mdpi.com/article/10.3390/ijms24021181/s1. Table S1. The Mammalian Phenotype report of the tested genes. Table S3. The enrichment analysis of genes in blue module. Figure S1: qPCR analysis of cytoskeletal components. Figure S2: Gene expression of extracellular matrix genes. Figure S3: Gene expression of focal adhesion molecules. Figure S4: Gene expression of growth factors. Figure S5: Gene expression of signaling factors. Figure S6: The sample cluster of the cancer cell lines. Figure S7: WGCNA network and module detection. Selection of the soft-thresholding powers. The left panel showed the scale-free fit index versus soft-thresholding power. The right panel displayed the mean connectivity versus soft-thresholding power. Figure S8: Clustering dendrogram of genes, with dissimilarity based on topological overlap, the red line indicates the cut height of 0.3, corresponding to merge correlation with 0.7. Figure S9: Bar graph of top 100 enriched terms across input gene lists, colored by *p*-values. Figure S10: Network of enriched terms colored by cluster ID, where nodes that share the same cluster ID are typically close to each other. Figure S11: Overview of raw Western Blots used in this study.

## Abbreviations

µ*g*	Microgravity
2D	Two-dimensional
3D	Three-dimensional
ACTB	Beta-actin
AD	Adherent monolayer
AKT1	RAC-alpha serine/threonine-protein kinase 1
CD44	Cell-surface glycoprotein
CDKN1	Cyclin-dependent kinase inhibitor 1A
CTNNA1	Catenin alpha-1
CTNNB1	Catenin beta-1
COL1A1	Collagen-1a1
COL4A5	Collagen-4a5
ECM	Extracellular matrix
EGF	Epidermal growth factor
EGFR1	Epidermal growth factor receptor 1
ERK1	Extracellular signal-regulated kinase 1
EVG	Elastica van Gieson staining
FAK1	Focal adhesion kinase 1 (PTK2)
FLK1	Fetal liver kinase 1
HARV	High aspect rotating-wall vessel
HE	Hematoxylin eosin staining
ITGB1	Integrin-beta-1
KDR	Kinase insert domain receptor
LAMA3	Laminin subunit alpha 3
MCS	Multicellular spheroid(s)
MAP2K1	Mitogen-activated protein kinase kinase 1
MAPK14	Mitogen-activated protein kinase 14
MTOR	Mechanistic target of rapamycin kinase
PAS	Periodic acid–Schiff staining
PCNA	Proliferating cell nuclear antigen
PFA	Paraformaldehyde
PIK3CB	Phosphatidylinositol-4,5-bisphosphate 3-kinase catalytic subunit beta
r-µ*g*	Real microgravity
RAF1	RAF proto-oncogene serine/threonine-protein kinase
RICTOR	Rapamycin-insensitive companion of mammalian target of rapamycin
RPM	Random positioning machine
RWV	Rotating wall vessel
SR	Sirius red staining
SRC1	Steroid receptor coactivator-1
s-µ*g*	Simulated microgravity
TUBB	Tubulin beta
VCL	Vinculin
VEGFA	Vascular endothelial growth factor A
