# Supplementary material for: Long-Term Simulation of Microgravity Induces Changes in Gene Expression in Breast Cancer Cells
Source: Int J Mol Sci. 2023 Jan 7;24(2):1181. doi: 10.3390/ijms24021181 (PMC9864731; doi:10.3390/ijms24021181)
Supplement: Supplementary file 1 [file ijms-24-01181-s001.zip › Supplemental Figures.pdf]

**Supplementary Figures**

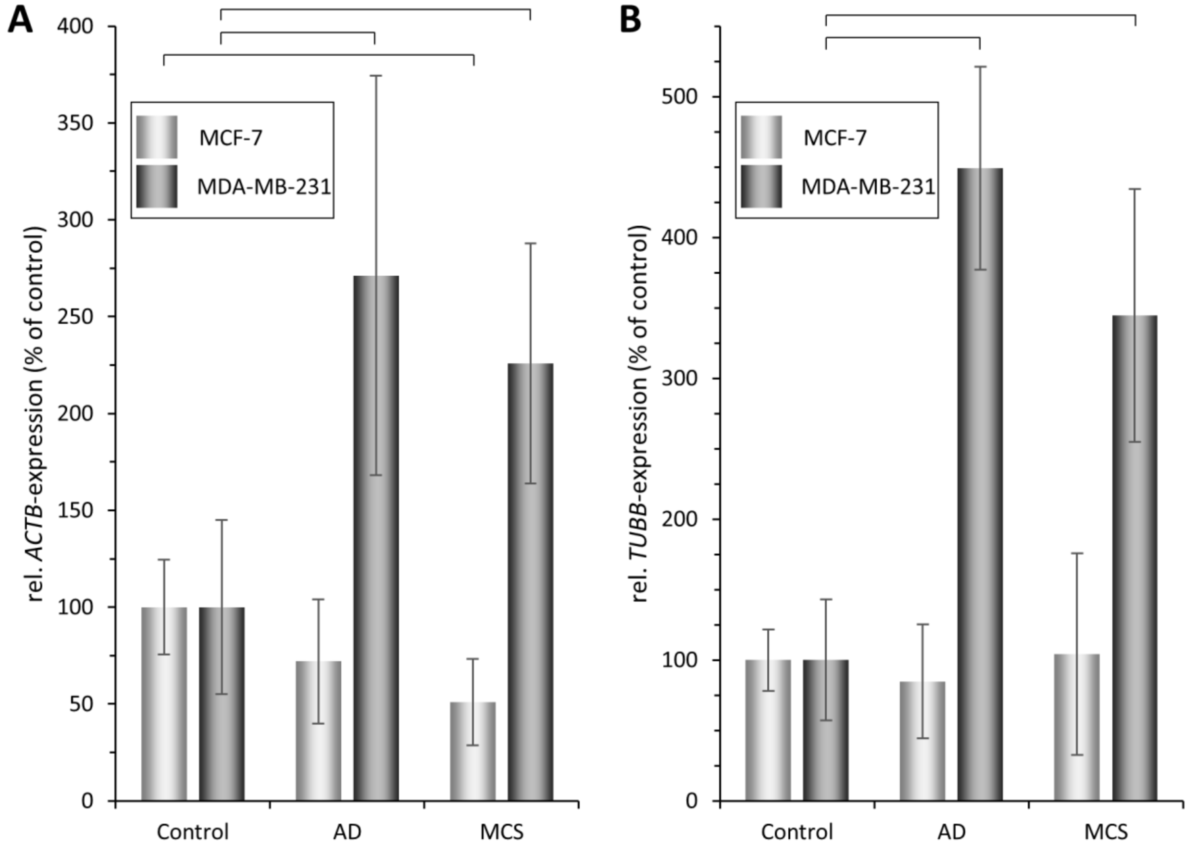


**Figure S1: qPCR analysis of cytoskeletal components**

**A:** gene expression of *ACTB*, **B:** gene expression of *TUBB*. n=5. Brackets indicate statistically significant differences with $P < 0.05$.

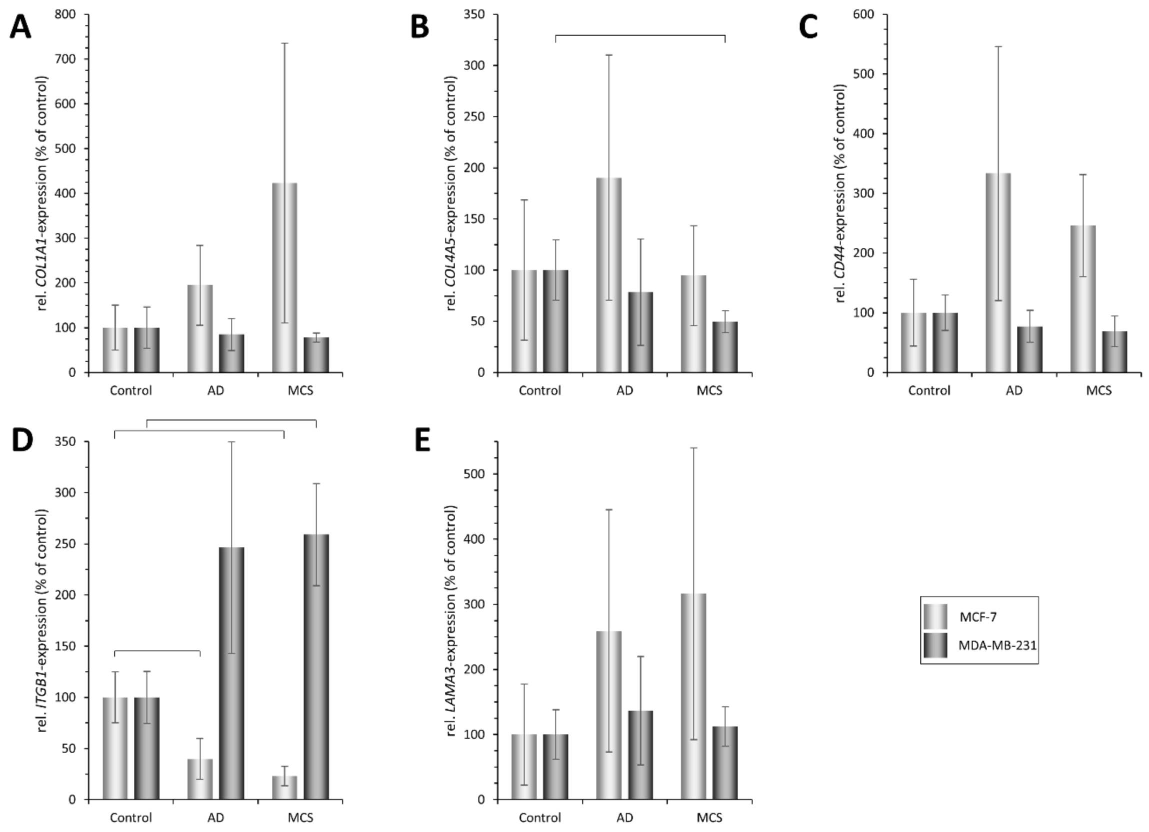


**Figure S2: Gene expression of extracellular matrix genes**

**A**: *COL1A1*, **B:** *COL4A5*, **C:** *CD44*, **D:** *ITGB1* and **E:** *LAMA3* gene expression profiles of MCF-7 and MDA-MB-231 BCC exposed to the RPM for 14d. n=5. Brackets indicate statistically significant differences with P < 0.05.

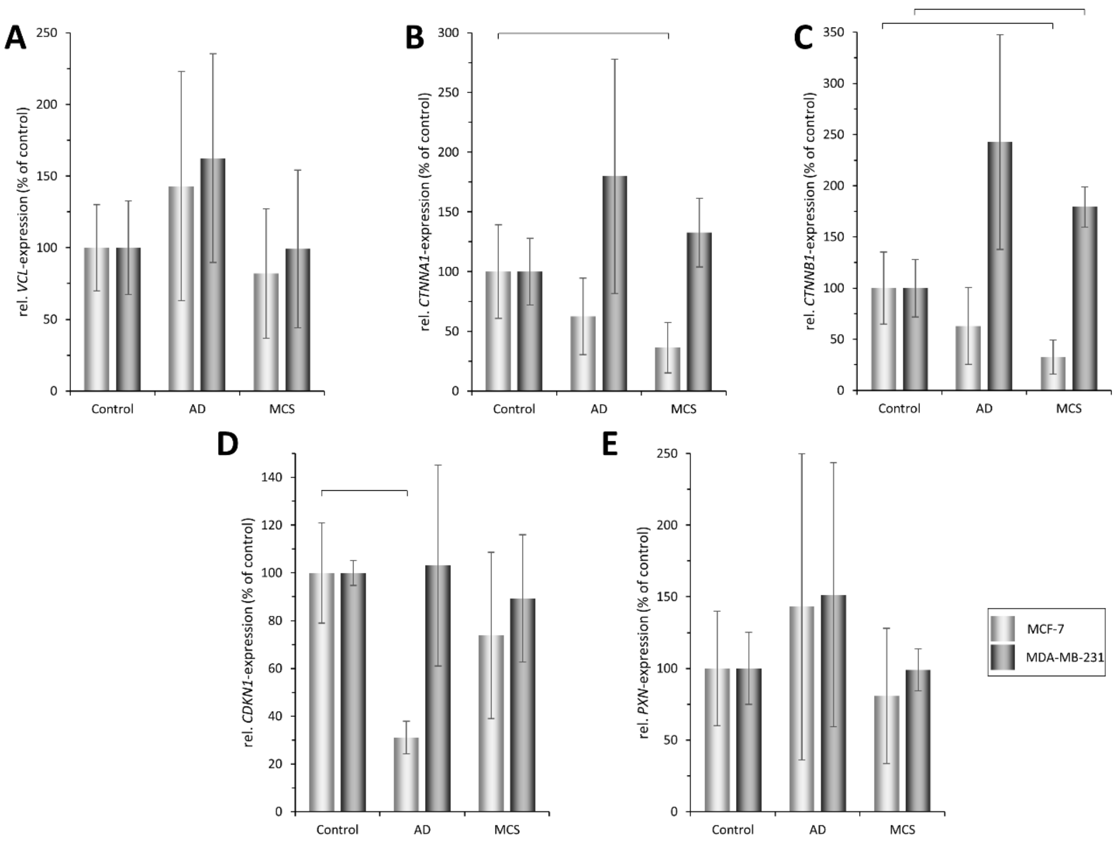


**Figure S3: Gene expression of focal adhesion molecules**

**A:** *VCL*, **B** *CTNNA1*, **C:** *CTNNB1*, **D:** *CDKN1*, and **E:** *PXN* gene expression profiles of MCF-7 and MDA-MB-231 BCC exposed to the RPM for 14d. n=5. Brackets indicate statistically significant differences with $P < 0.05$.

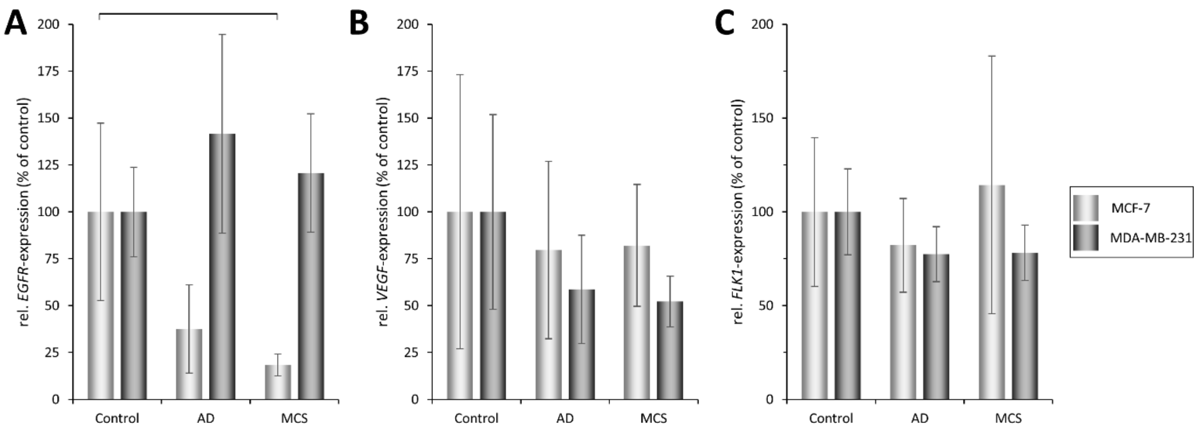


**Figure S4: Gene expression of growth factors**

**A:** *EGFR,* **B:** *VEGF,* and **C:** *FLK1* gene expression profiles of MCF-7 and MDA-MB-231 BCC exposed to the RPM for 14d. n=5. Brackets indicate statistically significant differences with $P < 0.05$.

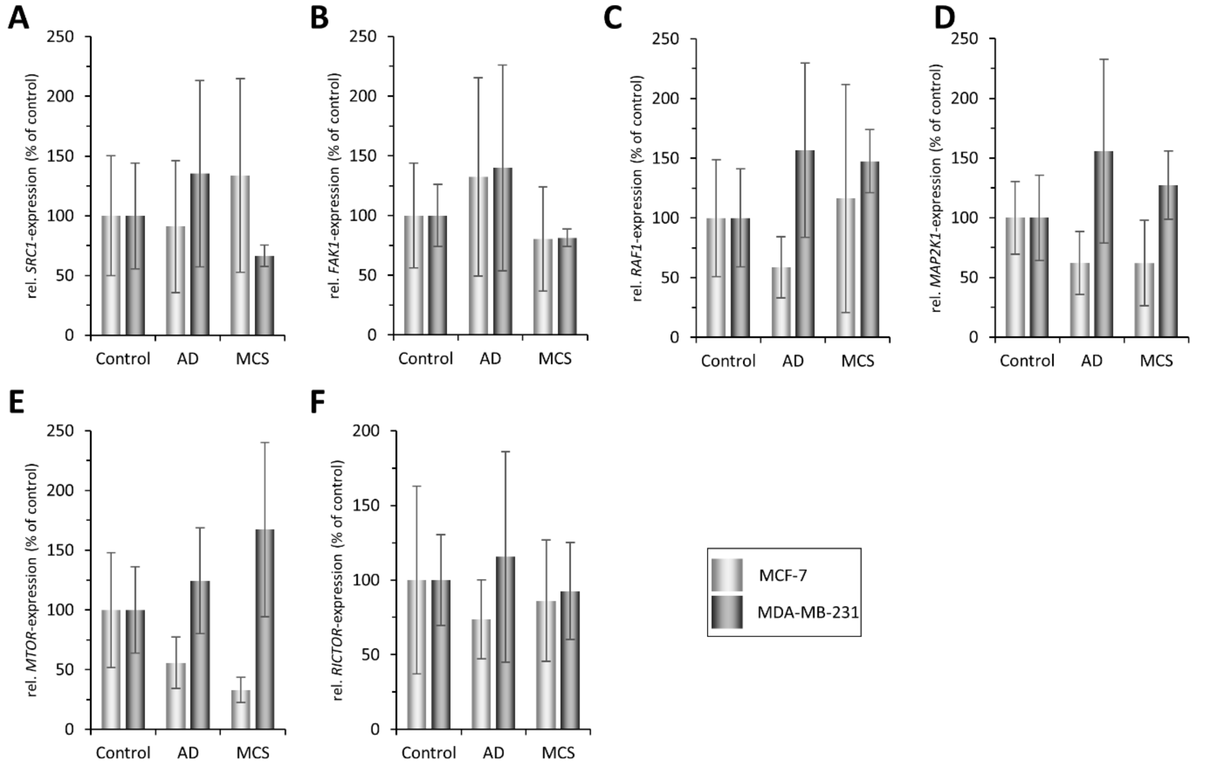


**Figure S5: Gene expression of signaling factors**

**A:** *SRC1,* **B:** *FAK1,* **C:** *RAF1,* **D:** *MAP2K1(MEK),* **E:** *MTOR* and **F:** *RICTOR* gene expression profiles of MCF-7 and MDA-MB-231 BCC exposed to the RPM for 14d. n=5. Brackets indicate statistically significant differences with $P < 0.05$.

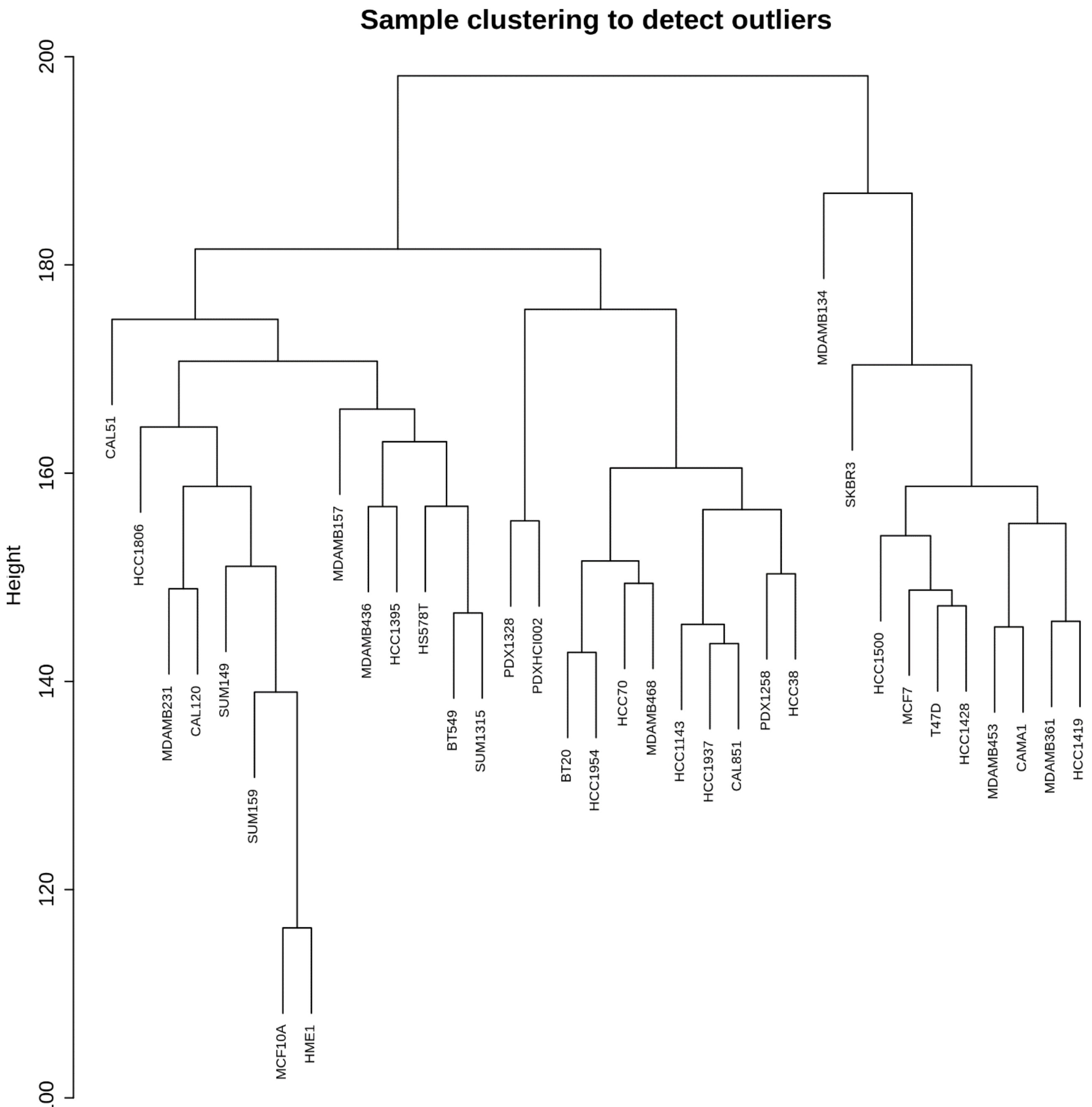


**Figure S6:** The sample cluster of the cancer cell lines.

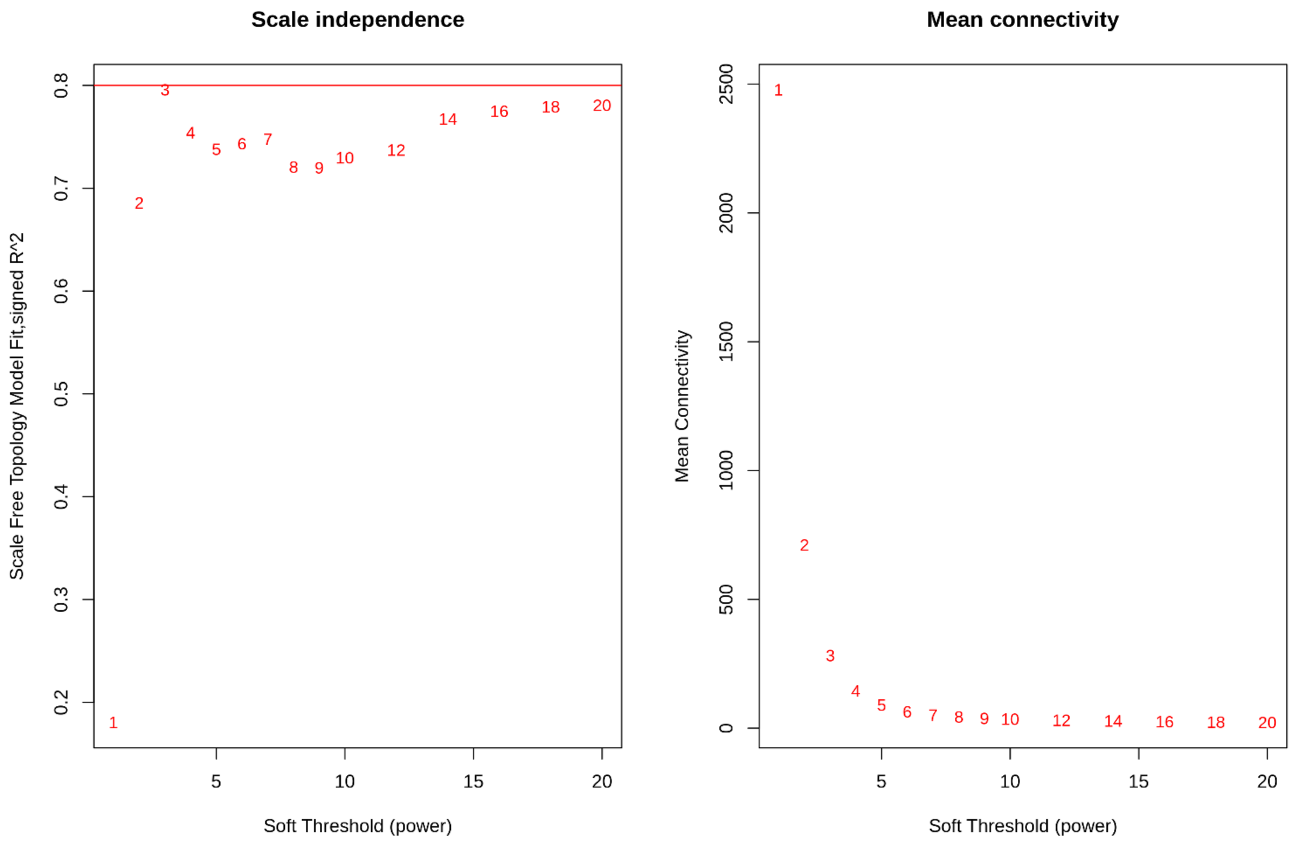


**Figure S7**: WGCNA network and module detection. Selection of the soft-thresholding powers. The left panel showed the scale-free fit index versus soft-thresholding power. The right panel displayed the mean connectivity versus soft-thresholding power.

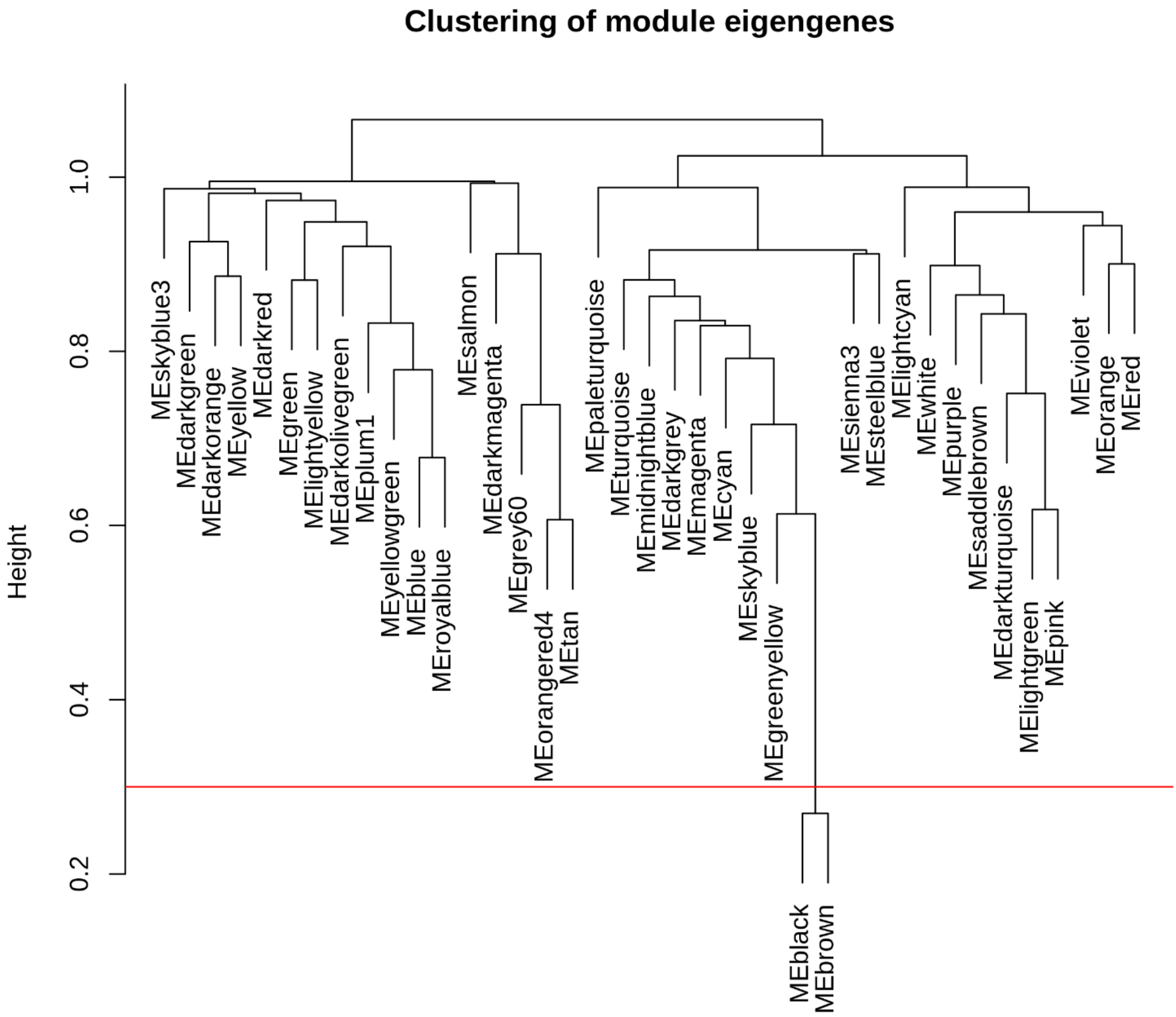


**Figure S8:** Clustering dendrogram of genes, with dissimilarity based on topological overlap, the red line indicates the cut height of 0.3, corresponding to merge correlation with 0.7.

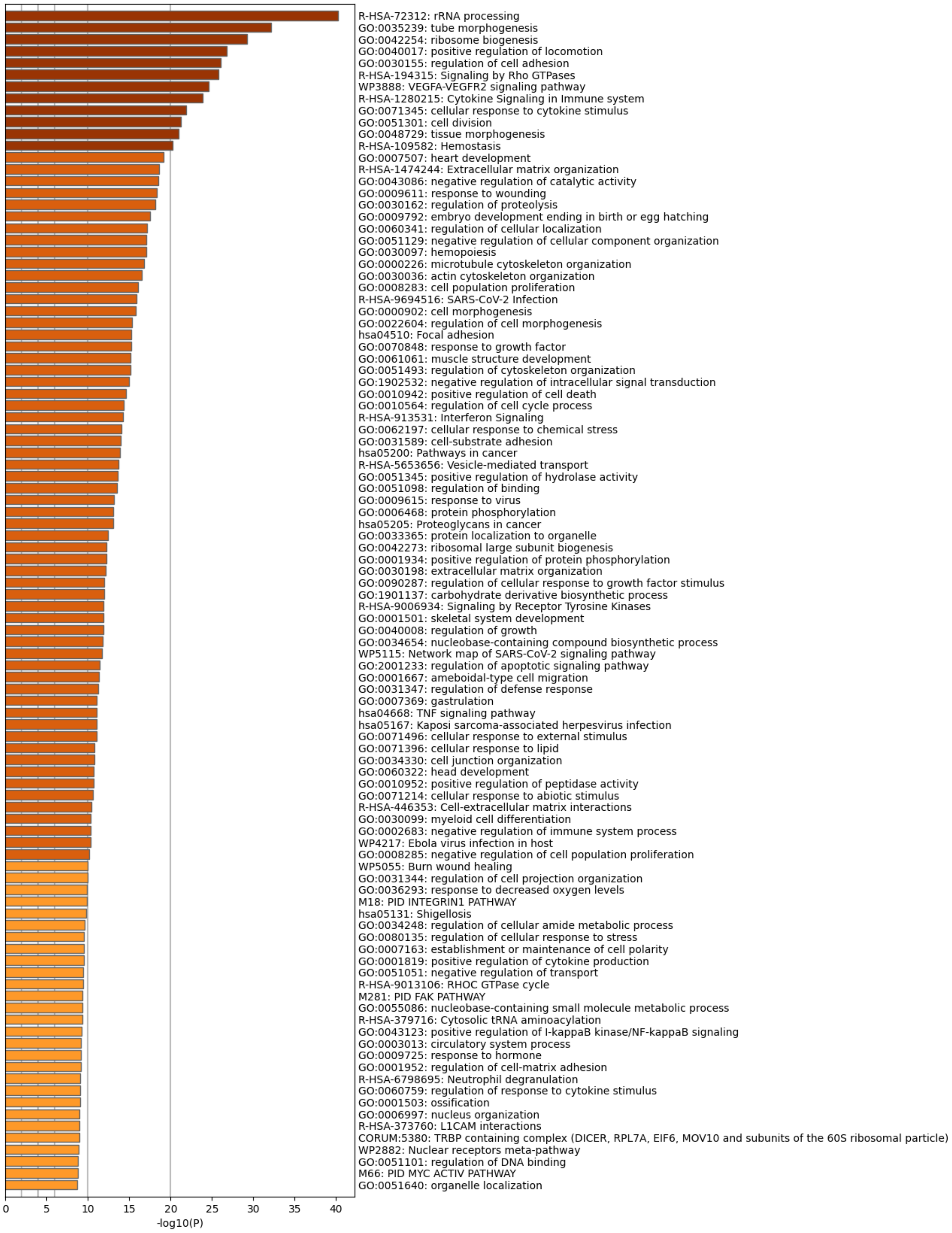


**Figure S9:** Bar graph of top 100 enriched terms across input gene lists, colored by p-values

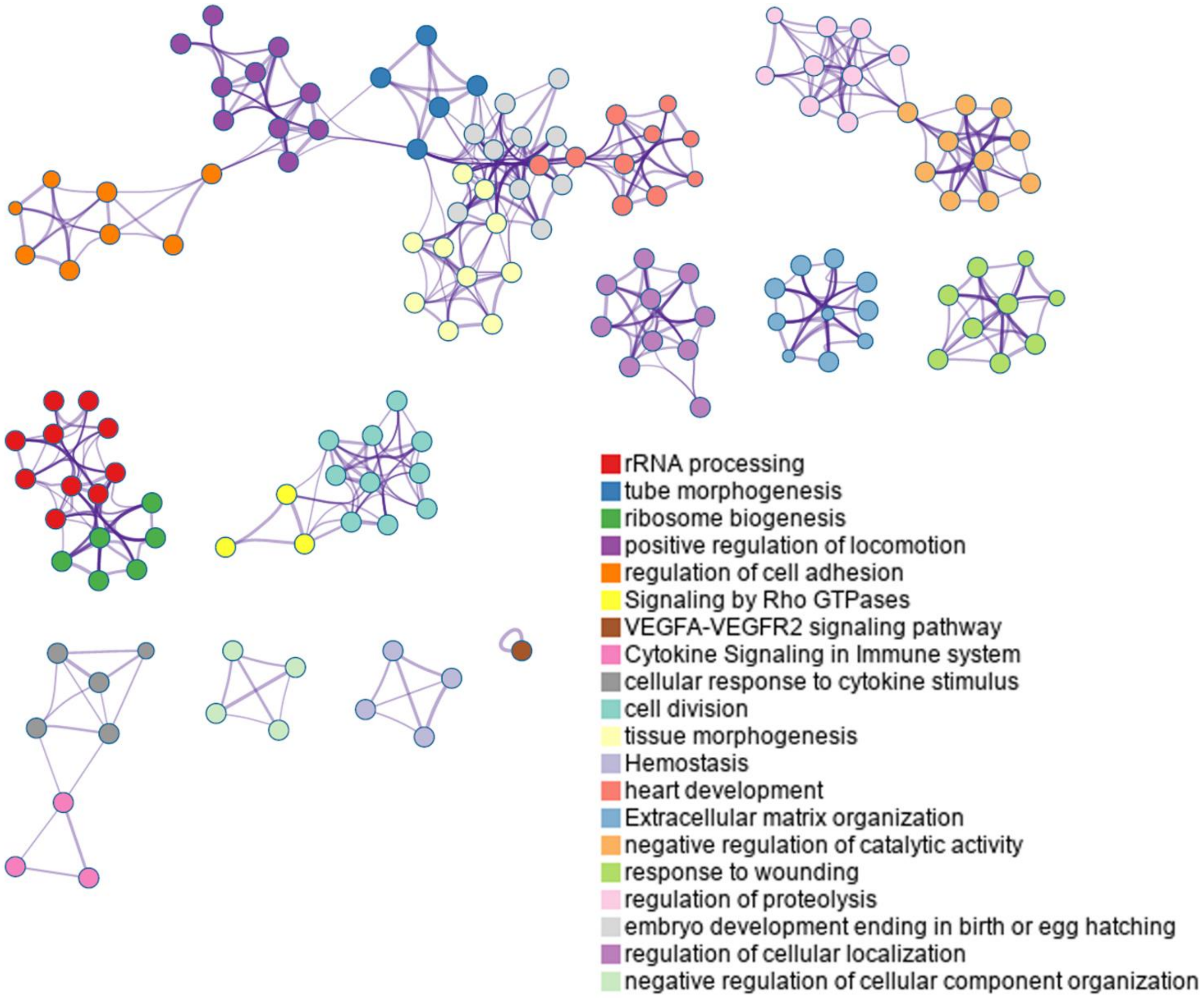


**Figure S10:** Network of enriched terms colored by cluster ID, where nodes that share the same cluster ID are typically close to each other.

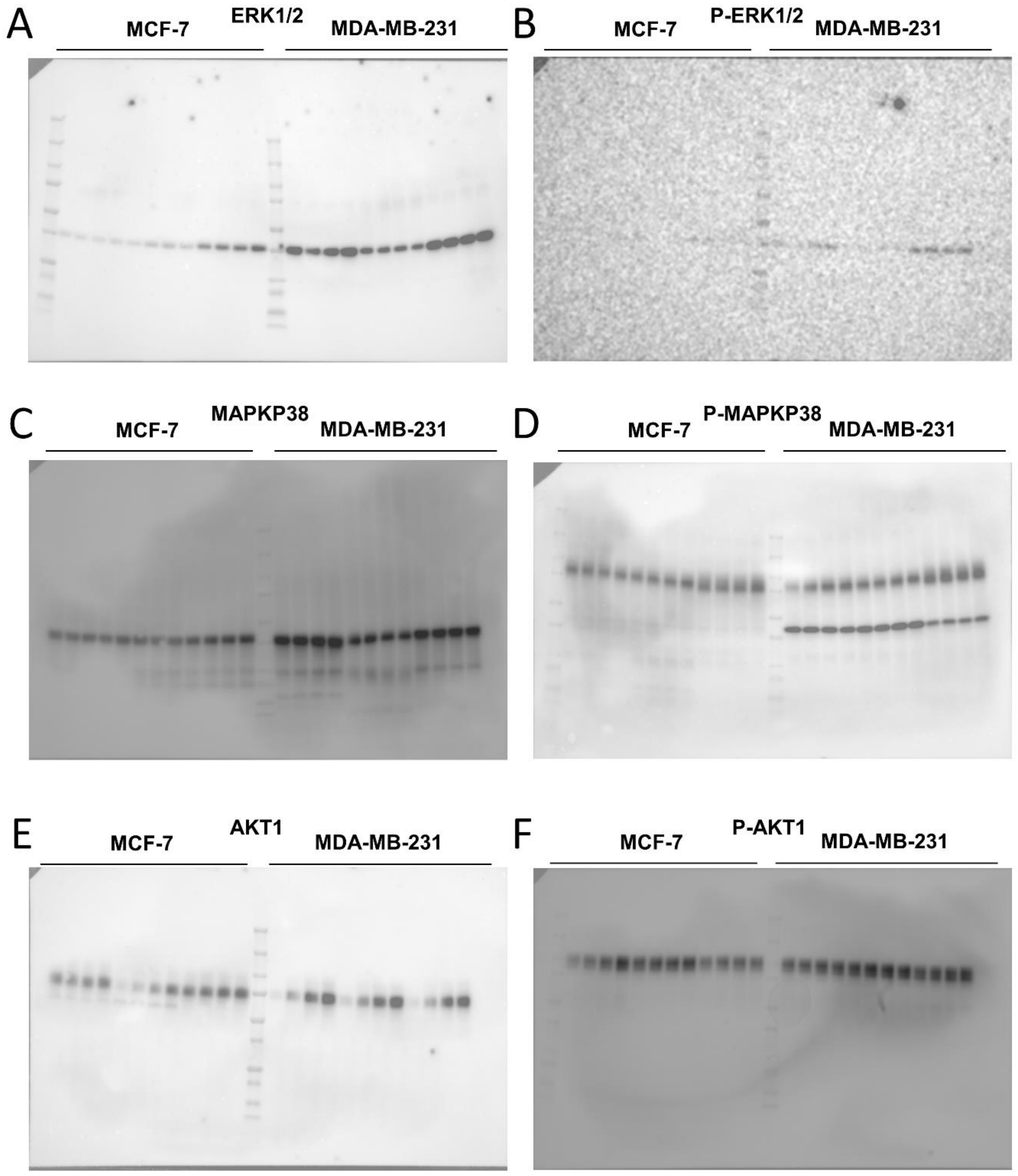


**Figure S11: Overview of raw Western Blots used in this study**.
